# Screening of Natural Product Derivatives Identifies Two Structurally Related Flavonoids as Potent Quorum Sensing Inhibitors against Gram-Negative Bacteria

**DOI:** 10.3390/ijms19051346

**Published:** 2018-05-03

**Authors:** Suvi Manner, Adyary Fallarero

**Affiliations:** 1 Pharmaceutical Sciences Laboratory, Faculty of Science and Engineering, Åbo Akademi University, Artillerigatan 6A, FI-20520 Turku, Finland; suvi.manner@abo.fi; 2 Pharmaceutical Design and Discovery (PharmDD), Pharmaceutical Biology, Division of Pharmaceutical Biosciences, Faculty of Pharmacy, University of Helsinki, Viikinkaari 5E, P.O. Box 56, FI-00014 Helsinki, Finland

**Keywords:** quorum sensing, quorum sensing inhibitor, *Chromobacterium violaceum*, natural product derivatives, flavonoids, *Pseudomonas aeruginosa*, *Escherichia coli*

## Abstract

Owing to the failure of conventional antibiotics in biofilm control, alternative approaches are urgently needed. Inhibition of quorum sensing (QS) represents an attractive target since it is involved in several processes essential for biofilm formation. In this study, a compound library of natural product derivatives (*n* = 3040) was screened for anti-quorum sensing activity using *Chromobacterium violaceum* as reporter bacteria. Screening assays, based on QS-mediated violacein production and viability, were performed in parallel to identify non-bactericidal QS inhibitors (QSIs). Nine highly active QSIs were identified, while 328 compounds were classified as moderately actives and 2062 compounds as inactives. Re-testing of the highly actives at a lower concentration against *C. violaceum*, complemented by a literature search, led to the identification of two flavonoid derivatives as the most potent QSIs, and their impact on biofilm maturation in *Escherichia coli* and *Pseudomonas aeruginosa* was further investigated. Finally, effects of these leads on swimming and swarming motility of *P. aeruginosa* were quantified. The identified flavonoids affected all the studied QS-related functions at micromolar concentrations. These compounds can serve as starting points for further optimization and development of more potent QSIs as adjunctive agents used with antibiotics in the treatment of biofilms.

## 1. Introduction

Biofilm formation complicates treatment of various infections, especially those related to the use of medical devices [1]. Conventional antibiotics aimed at killing or inhibiting the growth of dividing, planktonic cells are inefficient in the treatment of biofilm-associated infections. In biofilms, where cells exist as heterogeneous populations with various growth rates (including dormant cells), embedded in a protective matrix of extracellular polymeric substances, cell-to-cell communication plays an important role [2]. This process, termed quorum sensing (QS), seems to be crucial for biofilm formation, and it has emerged as a prominent target for finding effective strategies aimed at biofilm control.

Both Gram-negative and Gram-positive bacteria use QS to coordinate gene expression in a population density dependent manner. However, QS systems differ between species in terms of signal types, receptors, and signal transduction [3,4]. In Gram-negative bacteria, *N*-acyl-l-homoserine lactone (AHL) signal molecules called autoinducers (AIs) mainly mediate QS. In general, AHL-type AIs consist of a homoserine lactone (HSL) moiety couple with a fatty acid of varying length and oxidation state [5,6]. The AHL-dependent QS systems comprise of two proteins of LuxI and LuxR families, the former being responsible for the synthesis of the AIs and the latter detecting and binding of them [7]. The concentration of AIs increases in a cell density-dependent manner, and when a threshold concentration is reached, the signal molecules are bound to LuxR-like proteins. Thereafter, the AI–LuxR complex further activates the transcription of genes responsible for diverse phenotypes, such as violacein production in *Chromobacterium violaceum*, pathogenesis and virulence factors production, including motility and biofilm formation in *Pseudomonas aeruginosa* [4,8]. In *P. aeruginosa*, two AHL-dependent, LuxI/LuxR homologous systems, LasI/LasR and RhlI/RhlR exist. Moreover, the RhlI/RhlR system is under control of the LasI/LasR system at both transcriptional and posttranslational level [9].

Given the species specificity and broad-spectrum impact, QS represents an attractive target of anti-biofilm drug discovery. The inhibition of QS systems can be accomplished in different ways, namely, by inhibiting the signal generator, by degrading the signal molecule or by blocking the signal receptor [10]. The enzymatic degradation of the signal molecule is specifically known as quorum quenching [11]. Since QS regulates expression of several virulence factors, quorum sensing inhibitors (QSIs) can be used to attenuate bacterial virulence. Moreover, QSIs can make biofilms more susceptible to conventional antibiotics and the host immune system, and thus, lower doses and shorter antibiotic treatments would be needed [12,13]. Importantly, since QS does not affect the bacterial growth, QSIs are expected to be less prone to resistance development than bactericidal compounds [14].

Natural compounds have been a rich source of innovation in antimicrobial drug discovery, not only as antimicrobials but also as leads in drug design [15]. Natural compounds include complex structures that can provide a wide array of mechanisms of action compared to conventional antibiotics. Various types of QSIs have also been identified from natural sources [16,17]. Among the phytochemicals, halogenated furanones from the marine alga *Delisea pulchra* are the most studied class of QSIs [18,19,20]. In addition, diverse plant-derived compounds and their synthetic analogues and derivatives, such as tannins [21], cinnamaldehyde from cinnamon [22], iberin from horseradish [23], ajoene from garlic [24] and rosmarinic acid [25] have been reported to exhibit anti-quorum sensing activity against both Gram-negative and Gram-positive bacteria. 

In this context, the aim of this study was to identify QSIs from a library of 3040 chemically diverse, naturally inspired compounds. The library (NDL-3000, TimTec, Newark, DE, USA) containing natural derivatives, analogs, semi-natural compounds and mimics covers several compound classes, such as alkaloids, carbohydrates, flavonoids, steroidal compounds, amino acids, and purines. A microtiter well plate-based screening platform using *C. violaceum* as reporter bacteria was utilized for the identification of QSIs. The platform was recently optimized for the exploration of QSIs from natural compound libraries and successfully applied to the identification of flavonoids as QSIs by our group [26]. In *C. violaceum*, LuxI/LuxR homolog CviI/CviR is responsible for the production of violacein, a purple pigment, which serves as a useful indicator of QS. The loss of the pigment, in turn, indicates inhibition of QS [27]. Moreover, effects of the identified leads, selected based on the inhibition of violacein production and selectivity, were further evaluated against other QS-regulated functions, including biofilm maturation and motility in other Gram-negative species (*P. aeruginosa* and *Escherichia coli*), which use LuxI/LuxR homologs to control their communal behavior. Furthermore, effects of the leads on bacterial growth and cell viability were investigated. 

## 2. Results

### 2.1. Primary Screening for Quorum Sensing Inhibitors (QSIs) Using Two Strains of C. violaceum as Reporter Bacteria and Classification of the Compounds

**Figure 1:**
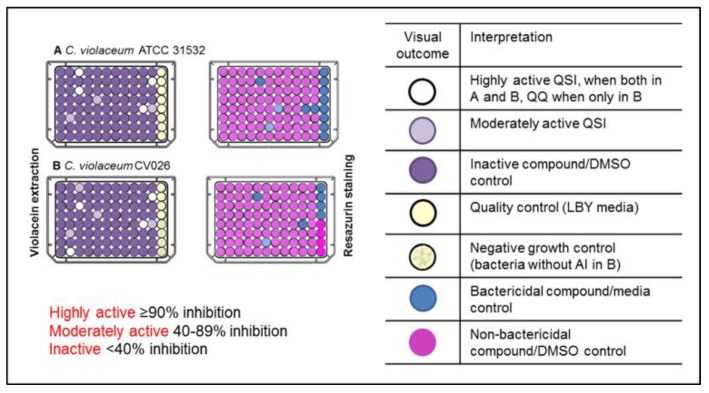
Screening for quorum sensing inhibitors. QSI = quorum sensing inhibitor, QQ = quorum quencher, AI = autoinducer, LBY = Luria–Bertani broth supplemented with yeast extract.

*C. violaceum* has been extensively used as a model bacterium in screening for QSIs [28,29,30,31,32,33]. Here, the compound library was initially tested against *C. violaceum*, utilizing screening assays for quantification of violacein production and viability in parallel, because only those compounds that induce inhibition of the violacein production, without affecting the growth of bacteria, can be considered as true QSIs [29]. Further, the simultaneous use of two strains of *C. violaceum*, American Type Culture Collection, ATCC 31532 and its mini-Tn5 mutant strain CV026 (National Collection of Type Cultures, Public Health England, NCTC 13278), as reporter bacteria, enabled the differentiation between QSIs and quorum quenchers (QQs), thus making the screening more comprehensive. This is because the mutant strain produces violacein only in the presence of exogenous AHLs [27], and therefore, it can be assumed that activity of those compounds, which were active only against this strain, is due to direct interference and degradation of AHL. Finally, QSIs were classified as highly actives, moderately actives and inactives according to the inhibitory activity on violacein production (Figure 1). 

**Figure 2:**
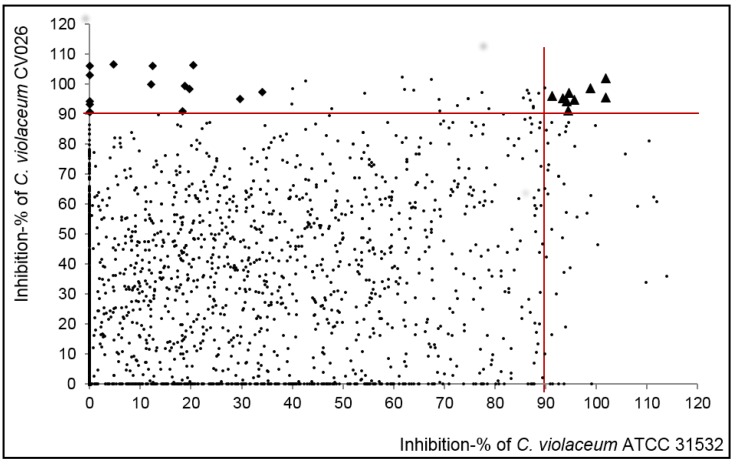
Inhibition of violacein production in *C. violaceum* ATCC 31532 and CV026 by the non-bactericidal compounds identified in primary screening (*n* = 2399). Highly active QSIs are marked with black triangles and QQs with black diamonds. Complete results of the primary screening are presented in Table S2. The red lines indicate the thresholds of 90% inhibition of violacein production, which were set to identify the highly active inhibitors from the rest of the screened compounds.

The selection criteria, applied to the classification of compounds, was adapted from our previous contribution [34]. To obtain a reasonable number of compounds for the secondary screens, the threshold for highly active compounds was set at ≥90% inhibition of violacein production, as compared to untreated controls on both strains. Altogether, 42 compounds, when tested at 400 µM, met this criterion. Among these compounds, 33 were found to display bactericidal activity (higher than 40% inhibition of bacterial viability) against one or both strains, as measured by resazurin staining assay. The remaining nine compounds were classified as highly actives, resulting in an overall hit rate of 0.3% (Figure 2). Only these compounds were selected for further studies. 

Additionally, 105 compounds exhibited strain-specific activity and inhibited violacein production by more than 90% in one strain with lower or no activity on the other. Of these, 73 compounds were non-bactericidal and classified as moderately actives. Further, from this group, 14 compounds were highly active against the mutant strain CV026 but inactive (less than 40% inhibition of violacein production) against ATCC 31532 strain, and they were categorized as QQs (*n* = 14) (Table S1). Moreover, all the compounds that resulted in an inhibition of 40–89% on violacein production without affecting the bacterial growth were also deemed moderately actives (*n* = 255). Finally, all the non-bactericidal compounds with less than 40% inhibitory activity on violacein production in one or both strains were classified as inactive (*n* = 2062). Altogether, 641 compounds displayed bactericidal activity. A complete list of the compounds included in the study (compound IDs, International Union of Pure and Applied Chemistry, IUPAC names and smiles, provided by TimTec, Newark, DE, USA, www.timtec.net, email: info@timtec.net) can be found in the Table S3. 

### 2.2. Highly Active QSIs 

Based upon the primary screening, nine highly active QSIs were identified (Table 1). 

Among these highly active compounds, flavonoids with five representatives (**341**, **575**, **916**, **2117** and **2896**) were the most represented class (Table 1). These plant secondary metabolites are one of the most widespread and extensively studied classes of natural products that have been shown to exhibit a number of biological activities [34,35,36]. Various flavonoids have also been reported with anti-QS activity against *C. violaceum*. Flavonols, such as quercetin [37,38,39] and kaempferol [37], glycosylated citrus flavanones naringin, hesperidin and neohesperidin [40], flavanones naringenin, eriodictyol and taxifolin [41], and flavan-3-ols, (−)-cathechin [42] and (−)-epicatechin [43] have shown to inhibit violacein production in *C. violaceum* without inhibiting the growth. Here, compounds **341**, **575**, **2117** and **2896** were characterized as flavonoid derivatives, more accurately, **341** as an isoflavonoid derivative, **575** as a flavonol derivative, and compounds **2117** and **2896** as flavone derivatives, whereas compound **916** was characterized as a naturally occurring flavone (6,7-dihydroxyflavone). To the best of our knowledge, the anti-QS activity of isoflavonoids against *C. violaceum* has not been previously reported. However, dalbinol, an isoflavonoid of rotenoid class, structurally-related to compound **341** has been shown to inhibit *P. aeruginosa* biofilms at low micromolar concentrations without antibacterial activity [44]. 

The four remaining highly active QSIs were identified as a nucleoside (cytidine) analogue (**144**), a mixture of two compounds (**339**, see details in Table 1), a lignan (**1698**) and an alkaloid (**2307**). Previously, QSI activity of some similar compounds, has been reported. Diverse nucleoside analogues have been demonstrated to interfere with auto-inducer 2 (AI-2)-based interspecies QS in multiple *Vibrio* species [45] but no previous reports exist either on cytidine analogues as QSIs or on inhibition of AHL-based QS-systems by nucleoside analogues. In **339**, a mixture of penicillin and 4-[[2-(diethylamino)ethoxy]methyl]aniline are present. Structurally distinct organosulfur compounds, such as isothiocyanates [43] and components of garlic extracts [46], have been identified as QSIs using *C. violaceum*. In addition, in **339**, the core structure of penicillin is included. Interestingly, ceftazidime, another antibiotic of beta-lactam class, has been shown to inhibit QS in *P. aeruginosa* at sub-MIC concentrations [47]. Compound **1698** was characterized as a furofuran type lignan, pinoresinol, glycosidically linked to a disaccharide, rutinose. Previously, anti-biofilm activity of another furofuran lignan, (+)-medioresinol, against *P. aeruginosa* and *E. coli* has been demonstrated [48], but so far, anti-QS activity against *C. violaceum* has not been reported, to the best of our knowledge. Compound **2307** comprises an aminothiazole linked to an indole moiety. Indole and indole-3-carbinol have demonstrated to inhibit violacein production in *C. violaceum* [49,50], and 4-(*o*-methoxyphenyl)-2-aminothiazole has been shown to act as a QSI in *P. aeruginosa* [51].

**Figure 3:**
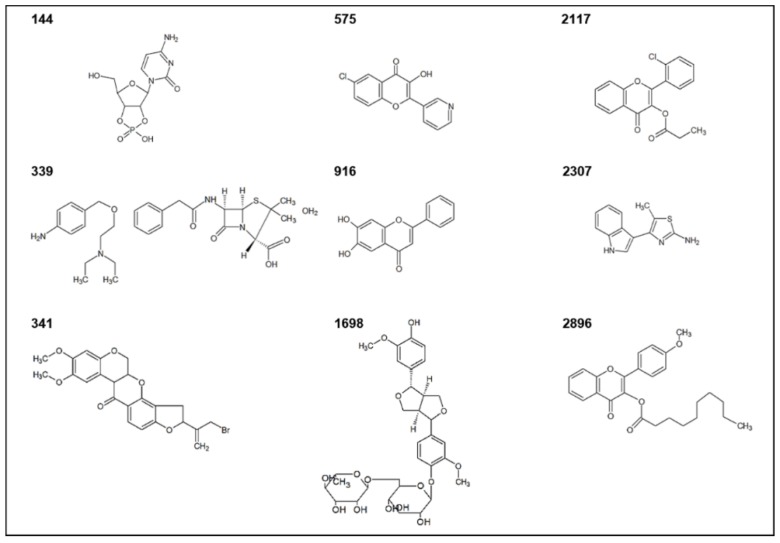
Structures of the highly active QSIs identified in the primary screening. The structures are drawn using ACD/ChemSketch software. The IUPAC names of the compounds are listed in Table S4.

Our group has previously reported the anti-QS activity of two of the compounds included within these highly active QSIs, in a screening study utilizing *C. violaceum* as reporter bacteria [26]. Flavone **916** was classified as moderately active, while compound **2117** was among the most active compounds. Furthermore, **916** has also been found to display anti-biofilm activity against *Staphylococcus aureus* by both preventing biofilm formation and by eradicating pre-formed biofilms [34]. These results demonstrate that compound **916** possess non-selective, broader anti-biofilm activity. Similarly, both anti-QS and anti-biofilm activities of other flavones, such as luteolin, apigenin and chrysin have been reported against both Gram-positive and Gram-negative bacteria [52,53,54]. Thus, this primary screening led to the identification of nine structurally diverse QSIs of *C. violaceum*, of which seven have not been previously reported. The structures of the highly active QSIs are shown in Figure 3. Notably, none of them fully resembles the structure of native AHLs in *C. violaceum* [55].

### 2.3. Selection and Characterization of the Leads

The effects of the highly active compounds on violacein production were measured at 40 µM against *C. violaceum* ATCC 31532. In this trial, only compound **2117** preserved its high activity by inhibiting violacein production by more than 90% (Table 2). Based on these results, compound **2117** and the second most active compound **2896**, which resulted in an inhibition of 83.5%, were selected as leads. In order to identify selective QSIs without any off-target effects, reported bioactivities associated with the highly active compounds were examined using PubChem Bioassay project database (http://www.ncbi.nlm.nih.gov/pcassay). No bioactivity records were found on the selected leads. While performing data mining on the leads, the other highly active compounds were also investigated. Two of them (**575** and **916**) were found to be included in previously reported studies. Compound **575** was not active, when investigated for the anti-cancer activity while compound **916** was found to display diverse bioactivities, including also inhibition of cancer cell lines [56]. This finding further emphasizes the multi-target mode of action of compound **916** instead of its potential to act as a selective QSI. 

Following the retesting, the half-inhibitory concentrations (IC_50_) of the two lead compounds (**2117**, **2896**) and quercetin, which was included as a positive control in primary screening, were determined against *C. violaceum* ATCC 31532. All the compounds exhibited concentration-dependent inhibition of violacein production. Potency (IC_50_) values were calculated using the Graphpad Prism software, and found to be in the low micromolar range (Table 2). Of the leads, compound **2117** had the lowest IC_50_ value of 9.6 µM.

**Figure 4:**
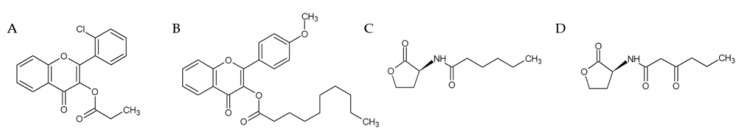
Structures of the lead compounds **2117** (**A**) and **2896** (**B**), *N*-hexanoyl-l-homoserine lactone (C6-HSL) (**C**), and autoinducer, *N*-(β-ketocaproyl)-l-homoserine used for *C. violaceum* CV026 cultures (**D**). The structures are drawn using ACD/ChemSketch software.

A clear structural similarity was observed between the two lead compounds **2117** and **2896** (see structures in Figure 4A,B). Both leads are flavonoid derivatives, in which esters of carboxylic (**2117**) and fatty (**2896**) acid are linked to the flavone backbone as side chains. However, they differ in the length of the side chain in addition to the substituents on the phenyl ring (B-ring). In **2117** (2-(2-chlorophenyl)-4-oxochromen-3-yl propanoate), chlorine atom is present, while **2896** (2-(4-methoxyphenyl)-4-oxochromen-3-yl decanoate) contains a methoxy group as a substituent (Figure 4).

Fatty acids have earlier been reported to interfere with QS and biofilm development in *P. aeruginosa*. For instance, lyngbyoic acid—a major metabolite of cyanobacterium *Lyngbya* cf. *majuscula*—has been shown to inhibit QS in *P. aeruginosa* [57]. Another example is the *cis*-2-decenoic acid—a fatty acid produced by *P. aeruginosa* that controls biofilms by both inhibiting biofilm formation and by inducing biofilm dispersal in both Gram-positive and Gram-negative species, including *P. aeruginosa* [58]. It may then be speculated that the presence of a fatty acid promotes the anti-QS activity of the compound **2896**. Moreover, a slight structural similarity among the two identified leads and two different AHL can be seen (Figure 4). The carbonyl function is located at the first carbon in the side chain. These AHLs are the *N*-hexanoyl-l-homoserine lactone C6-HSL, the short chain AHL produced by *C. violaceum* ATCC 31532 [59] and the autoinducer (*N*-(β-ketocaproyl)-l-Homoserine) added to *C. violaceum* CV026 cultures to induce violacein production. Thus, it can be hypothesized that the lead compounds may act as QS-inhibitors by competing with AHLs for receptor binding to the LuxR homologue, in a manner resembling competitive inhibition. Furthermore, in the CV026 strain, which requires exogenous AHL (C4 to C8) to produce violacein, addition of AHLs with longer side chains (C10 to C14) is known to lead to inhibition of violacein production [27]. This fact may further explain the activity of the compound **2896**. 

Finally, it is important to highlight that both leads were included in the flavonoid library, previously screened by our group for anti-biofilm activity against *S. aureus* [34]. In this study, both compounds were classified as inactives. This further supports the presented evidence that the compounds **2117** and **2896** preferentially act only on Gram-negative bacteria and, therefore, emphasizes their potential as selective QSIs.

### 2.4. Impact of the Leads on Biofilm Maturation and Architecture in Other Gram-Negative Species

**Figure 5:**
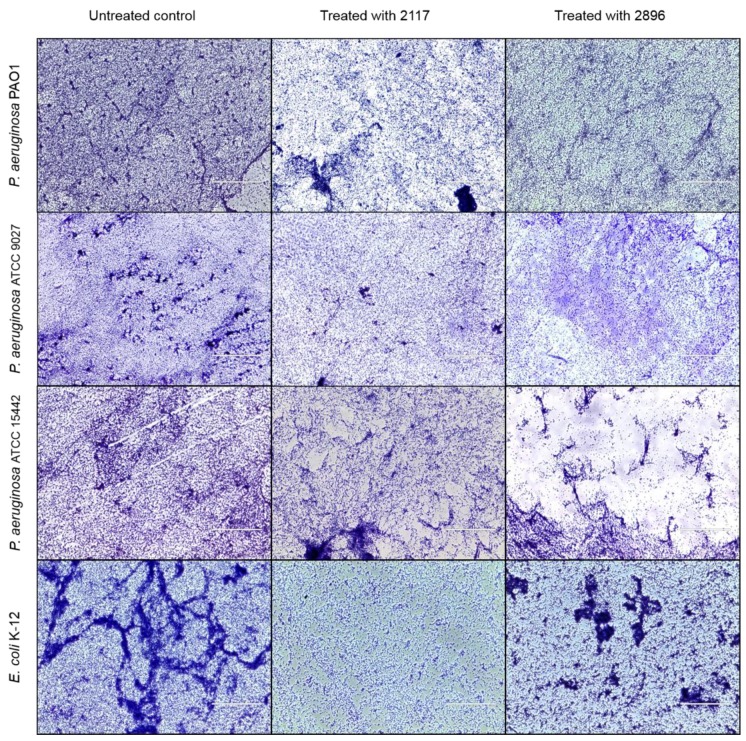
Transmitted light microscopy images of *P. aeruginosa* and *E. coli* biofilms grown in the absence and presence of the lead compounds. Biofilms are stained with crystal violet and visualized at a magnification of 40×. Scale bars correspond to 100 µm.

Since QS plays a significant role in biofilm formation and differentiation [9,60] effects of the leads were studied on the maturation of several *P. aeruginosa* strains (ATCC 9027, ATCC 15442 and PA01) as well as *E. coli* K-12 biofilms. In *P. aeruginosa*, LuxI/LuxR homolog LasI/LasR system regulates biofilm maturation [61], while in *E. coli*, orphan LuxR homolog sdiA [62] is likely involved in biofilm maturation. Indeed, compounds inhibiting CviI/CviR system in *C. violaceum* have also shown to interfere with biofilm maturation of *E. coli* [26]. In this assay, effects of the compounds **2117** and **2896** on the transition from microcolonies to fully formed biofilms were investigated using crystal violet staining and transmitted light microscopy. The microscopic analysis revealed that exposure to the lead compounds at 100 µM led to the alteration of biofilm structure and reduced biofilm formation when compared to untreated biofilms of all the tested strains (Figure 5). However, the impact varied between compounds and strains. Qualitative changes in biofilm architecture have been also reported by other QSIs, such as halogenated furanones and garlic [63,64]. 

Further, quantitative analysis showed that biofilm biomass was reduced by 11–51% compared to the untreated biofilms, when formed microcolonies were exposed and incubated with the lead compounds (Table 3). 

Compound **2117** displayed higher inhibitory activity than **2896**, except for in case of *P. aeruginosa* ATCC 15422. These results are in line with the observations made on biofilm architecture (Figure 5). However, overall, the inhibitory activity of the leads even at the highest test concentration (400 µM) against these Gram-negative species remained modest as compared to their activity against *C. violaceum*. This finding can be likely explained by the fact that even though the QS-systems of *C. violaceum* and *P. aeruginosa* are homologs of LuxI/LuxR system, they differ between species. Strain-specificity, over the system-specificity has been reported for QSIs [23]. 

Nevertheless, these observations demonstrate the potential of the leads to disrupt biofilm maturation leading to formation of weaker biofilms. Therefore, it can be proposed that use of the leads as adjunctive agents could increase the antimicrobial susceptibility of biofilms, and further enhance the efficacy of the conventional antibiotics. Such features have been previously reported for QSIs [12,63,65]. 

### 2.5. Impact of the Leads on Swarming and Swimming Motility

**Figure 6:**
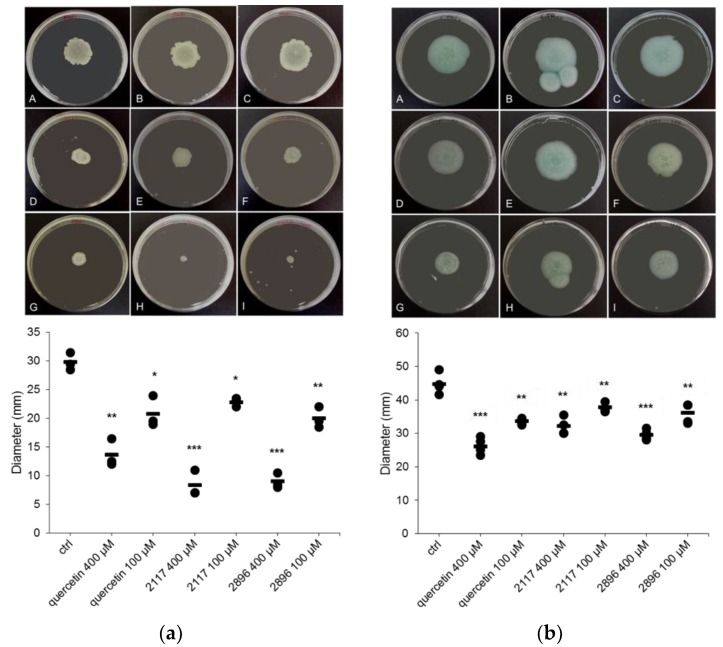
Inhibition of swarming (**a**) and swimming (**b**) motility of *P. aeruginosa* PAO1 by the lead compounds. In both panels, (**A**–**C**) untreated *P. aeruginosa* PAO1, (**D**–**F**) *P. aeruginosa* PAO1 treated with quercetin, **2117** and **2896** at 100 µM, and (**G**–**I**) *P. aeruginosa* PAO1 treated with quercetin, **2117** and **2896** at 400 µM, respectively. Results are shown as mean ± SD of three (swarming) and four (swimming) independent experiments. Statistical differences are marked in the Figure as: * *p* < 0.05; ** 0.001 < *p* < 0.05 and *** *p* < 0.001).

QS-mediated swarming and swimming motilities are considered as important features of Gram-negative bacteria for the surface attachment during the early steps of biofilm formation as well as for biofilm maturation [66,67,68]. Therefore, the ability of the lead compounds to interfere with these processes in *P. aeruginosa* PAO1 was studied. Various compounds, for example, phenolic compounds, such as flavonol quercetin [37,39], flavones chrysin and baicalein [69], and proanthocyanidins from cranberry [70] have been shown to inhibit motility of *P. aeruginosa*. Moreover, a fatty acid, *anteiso*-C_15:0_ has been also found to inhibit swarming motility of *P. aeruginosa* [71]. Here, the leads significantly reduced both types of motilities in a concentration-dependent manner, as illustrated in Figure 6. When tested at the highest test concentration (400 µM), both leads **2117** and **2896** were more effective compared to quercetin, used as a positive control in the assay, by inhibiting swarming motility by 72.1% and 69.8%, respectively. In terms of swimming motility, higher inhibition (33.9%) was recorded for the compound **2896** than compound **2117** (28.1%). Inhibition of motility can be correlated with the reduced capacity of *P. aeruginosa* to form biofilms in presence of the leads. However, the lead compounds did not affect the RhlI/RhlR controlled rhamnolipid biosynthesis in *P. aeruginosa* that has been connected to swimming and swarming motility [72,73].

### 2.6. Impact of the Leads on Growth of P. aeruginosa and E. coli 

To confirm that any of the observed anti-QS activities was not connected to bactericidal activity, impact of the leads on the growth of *P. aeruginosa* and *E. coli* was quantified. Neither of the leads displayed any bactericidal activity, as determined by viable plate counts (Figure S1). Hence, the identified lead compounds **2117** and **2896** can be considered as true QSIs that do not rely upon antibacterial activity as conventional antimicrobials [13].

### 2.7. Cytotoxicity of the Leads 

In vitro cytotoxicity of the leads was measured against HL and RAW 264.7 cell lines. Compound **2117** when assayed at the highest test concentration (100 µM) affected the viability of both cell lines to varying extent, while compound **2896** did not display any effects on the cell viability after 24 h exposure (Table 4). A clear cytotoxic effect of **2117** was observed against RAW 264.7 cell line, whereas against HL cells it demonstrated minor cytotoxicity (<20% inhibition of the viability). Thus, results obtained from the cytotoxicity assay indicate that compound **2896**, without any off-target cytotoxicity, is more selective than **2117** as a QSI. However, **2117** inhibited QS in *C. violaceum* at substantially lower, non-toxic concentrations.

## 3. Materials and Methods

### 3.1. Compound Library 

A Natural Derivatives Library, NDL-3000 composed of 3040 distinct samples was purchased from TimTec Inc. (Newark, DE, USA). Compounds in the library were dissolved in dimethyl sulfoxide (DMSO, minimum 99.9%; Sigma-Aldrich, St. Louis, MO, USA) to a concentration of 20 mM, and stored in matrix tubes at −20 °C. Purity (>95%) and identity of the compounds was confirmed by the supplier using High-Performance Liquid Chromatography (HPLC), and Nuclear Magnetic Resonance spectroscopy (NMR) (300 MHz or higher) and Liquid Chromatography-Mass Spectrometry (LC/MS), respectively. The NDL-3000 library comprises chemically diverse semi-natural, natural derived, and natural compounds-like synthetic compounds, such as alkaloids, natural phenols, nucleoside analogs, carbohydrates, purines, pyrimidines, flavonoids, steroidal compounds and natural amino acids (http://www.timtec.net). A complete list of the compounds included in the study (compound IDs, IUPAC names and smiles as provided by TimTec, Newark, DE, USA, www.timtec.net, email: info@timtec.net) is included in Table S3. 

### 3.2. Bacterial Strains, Media and Culture Conditions

*C. violaceum* (ATCC 31532, a biomarker strain) and *P. aeruginosa* (ATCC 9027, ATCC 15442) were purchased from American type Culture Collection (ATCC; Wesel, Germany) and *C. violaceum* CV026 (NCTC 13278, a mini-Tn5 mutant of *C. violaceum* ATCC 31532) from Public Health England’s National Culture of Type Collection (NCTC; Salisbury, UK). *P. aeruginosa* PAO1 and *E. coli* K-12 were obtained from University of Helsinki, HAMBI collection (http://www.helsinki.fi/hambi/). 

*C. violaceum* was grown in 3 mL of 30 g/L tryptic soy broth (TSB, Fluka Biochemika, Buchs, Switzerland) until an optical density 595 (OD_595_) equivalent to 0.7 (~10^9^ CFU/mL) under aerobic conditions (27 °C, 200 rpm shaking) overnight. *P. aeruginosa* (ATCC 9027, ATCC 15442 and PA01) and *E. coli* K-12 were pre-cultured in 3 mL of 20 g/L Luria–Bertani broth (LB, Serva Electrophoresis GmbH, Heidelberg, Germany) under aerobic conditions (37 °C, 220 rpm shaking) overnight, followed by 100-fold dilution in 10 mL of LB, and further incubation under aerobic conditions (37 °C, 200 rpm shaking) to an OD_595_ of ~0.4 (approximately 10^8^ CFU/mL). Optical densities were measured using Varioskan Flash Multimode Plate Reader operated with SkanIt RE for Varioskan Flash 2.4.3 software (Thermo Scientific Oy (Vantaa, Finland), and CFUs were confirmed by serial dilution of cultures and plating on tryptic soy agar (TSA, Fluka Biochemika, Buchs, Switzerland) (*C. violaceum*) and Luria–Bertani (LB) agar Miller (Fisher Scientific, Leicestershire, UK) (*P. aeruginosa* and *E. coli*). 

### 3.3. Screening for Quorum Sensing Inhibitors (QSIs)

The initial screening of compound library was conducted using two strains of *C. violaceum* as model bacterium, and two assays (violacein extraction and resazurin staining) in parallel as recently optimized in [26]. For the screening assays, overnight cultures of *C. violaceum* were diluted in LB broth supplemented with yeast extract (LBY) (0.1% *w*/*v*, Sigma Aldrich, St. Louis, MO, USA). Culture of *C. violaceum* CV026 was further supplemented with 0.5 µM *N*-(β-ketocaproyl)-l-Homoserine lactone (3-*O*-C_6_-(l)-HSL, Cayman chemicals, Ann Arbor, MI, USA) to induce the violacein production. Bacteria (10^6^ CFU/mL) was exposed to compounds (400 µM) in a total volume of 200 µL in 96-microtiter well plates (Nunc, Roskilde, Denmark), and incubated at 27 °C, with shaking at 200 rpm for 24 h. DMSO was added in untreated control wells and bacteria-free wells with only LBY were included controls. Additionally, autoinducer-free wells containing only bacteria were included in *C. violaceum* CV026 plates. Plates were prepared in duplicates. Quercetin dihydrate (Carl Roth GmbH, Karlsruhe, Germany) at 400 µM was used as positive control in violacein extraction assay, and azithromycin (Cayman chemicals, Ann Arbor, MI, USA) at 10 µM in viability assay. Both control compounds were prepared in DMSO. DMSO concentration was 2.5% throughout the experiments. 

#### 3.3.1. Violacein Extraction

After incubation period of 24 h, plates were centrifuged (Allegra™ X-12R Benchtop centrifuge, Beckman Coulter Inc., Fullerton, CA, USA) at 3000 rpm for 10 min to precipitate the violacein. Supernatants were discarded from the wells by pipetting, and pellets were re-suspended in 200 μL of 96% ethanol by scraping and mixing followed by centrifugation (3000 rpm, 10 min). Thereafter, 100 μL of supernatants were transferred to sterile 96-microtiter well plates, and violacein was quantified by reading the optical density at 595 nm using Varioskan Flash. Inhibition of violacein production was expressed as inhibition percentages of the untreated biofilms (Equation (1)):

Inhibition-% = [(untreated control OD_595_ − sample OD_595_)/(untreated control OD_595_ − media control OD_595_)] × 100% (1)

in which untreated control = *C. violaceum* + DMSO, media control = LBY, and sample = *C. violaceum* treated with a compound. Later on, violacein extraction assay was also utilized for the determination of half inhibitory concentrations of the compounds.

#### 3.3.2. Resazurin Staining Assay

Effects of the compounds on bacterial viability were quantified using resazurin staining assay. After the 24 h incubation, plates were centrifuged (3000 rpm, 10 min) and the supernatants were removed from the wells using multichannel pipette. Resazurin solution (20 µM) prepared in phosphate buffered saline (PBS, Lonza, Viewers, Bornem, Belgium) was added to the wells (200 μL per well), and plates were incubated in darkness, at room temperature (RT) with shaking at 200 rpm for 45 min. Fluorescence (λ_exc_ = 560 nm; λ_em_ = 590 nm) was read using Varioskan Flash. Bactericidal activity of the compounds was assessed as inhibition percentage of biofilm viability (Equation (2)):

Inhibition-% = [(untreated control − sample)/(untreated control − media control)] × 100%(2)

### 3.4. Crystal Violet Staining Assay

Crystal violet-based biomass staining assay was performed to assess the effects of the compounds on the transition from microcolonies to fully formed as recently described in [26] with minor modifications. Briefly, diluted cultures of exponentially grown *P. aeruginosa* ATCC 9027, ATCC 15442 and PA01, and *E. coli* K-12 (10^6^ CFU/mL) were added to the 96-microtiter well plates (200 µL per well) to form microcolonies for two hours (37 °C). At the end of this period, bacterial suspensions were removed, compounds at a final concentration of 400 µM and fresh LB media were added, and plates were incubated at 37 °C, with shaking (200 rpm) for additional 22 h. After the incubation period, media was discarded and the biofilms were washed once with sterile Milli-Q (MQ)-water. Biomass was fixed with methanol for 15 min followed by staining with 0.23% (*v*/*v*) crystal violet (Sigma-Aldrich, Munich, Germany) solution prepared in MQ-water (200 µL per well) for 5 min. Thereafter, wells were washed twice with MQ-water and 200 µL of 96% ethanol was added to the wells. Absorbance was measured at 595 nm using Varioskan Flash after 1 h. 

### 3.5. Transmitted Light Microscopy

For imaging, bacteria were allowed to adhere and form microcolonies on coverslips. Briefly, 5 mL of diluted cultures (10^6^ CFU/mL) of *P. aeruginosa* and *E. coli* was added to 6-well plates containing glass coverslips (22 mm × 22 mm). The plates were incubated at 37 °C without shaking for 2 h. Thereafter, bacterial suspensions were removed, and the wells were washed once with PBS. Fresh LB with and without the lead compounds at a final concentration of 100 µM was added to the wells, and the plates were incubated at 37 °C for 22 h. At the end of 24 h, coverslips were rinsed three times with MQ-water and air dried followed by staining with 0.23% (*v*/*v*) crystal violet solution. Coverslips were allowed to dry and visualized under a Transmitted Light Microscope (EVOS^®^ XL Imaging System, InvitrogenTM, Thermo Fisher Scientific, San Jose, CA, USA) at a magnification of 40×.

### 3.6. Swarming and Swimming Motility Assays

Assays were performed according to the protocols previously described in [30,74] with some modifications. The swarming agar was prepared in MQ-water consisting of 0.3% (*w*/*v*) agar, 1% (*w*/*v*) peptone, 0.5% (*w*/*v*) yeast extract and 0.5% (*w*/*v*) sodium chloride, and the swimming agar similarly but without yeast extract. Compounds at a final concentration of 400 µM and 100 µM were pre-mixed with 5 mL of agar, and poured over solidified LB agar plates (10 mL/petri dish) as overlays. The plates were allowed to dry at 30 °C for three hours followed by point inoculation with 2 µL of *P. aeruginosa* PAO1 (~10^8^ CFU/mL) and incubation at 37 °C for 18 h. Quercetin was used as a control. Diameters of the swarming and swimming zones were measured after the incubation periods.

### 3.7. Viable Plate Counts

Effects of the leads on bacterial growth were assessed by performing viable plate counts. Compounds at final concentrations of 100 and 400 µM and cultures of *P. aeruginosa* (ATCC 9027, ATCC 15422 and PA01) and *E. coli* K-12 (10^6^ CFU/mL) were simultaneously added to the 96-microtiter well plates (200 µL per well). Untreated bacteria were included in control wells. The plates were incubated at 37 °C, without shaking for 24 h. At the end of this period, bacterial suspensions were transferred from the wells to Eppendorf tubes, and the tubes were sonicated for 10 min (25 °C, 35 kHz, Sonorex Digitec water bath (Bandelin, Zurich, Switzerland)). Each suspension was serially diluted in LB and plated on LB agar. The number of viable bacteria in each test concentration was counted after overnight incubation at 37 °C. Results were expressed as CFU/mL.

### 3.8. Cytotoxicity Testing

Human lung (HL) epithelial cells [75] were grown in RPMI 1640 (Biowhittaker, Lonza, Viewers, Bornem, Belgium) supplemented with 10% inactivated fetal bovine serum (FBS), 2 mM l-glutamine and gentamycin 20 µg/mL, and mouse monocyte macrophage RAW 264.7 cells in DMEM medium supplemented with 10% FBS and 20 µg/mL gentamycin. Cells (3 × 10^5^ cells/mL) were seeded into 96-microtiter well plates (Cellstar TC, Greiner Bio-One GmbH, Frickenhausen, Germany), 200 µL per well, and incubated at 37 °C in 5% CO_2_ 24 h before exposure to the compounds. After the incubation, culture medium was discarded, the wells were washed with PBS, and the compound dilutions prepared in culture media were added as triplicates to a final volume of 200 µL. Usnic acid (200 µM, Sigma Aldrich, St. Louis, MO, USA) was included as positive control and DMSO (1%) as solvent control. Plates were incubated for additional 24 h. At the end of 24 h, media was removed, wells were washed twice with PBS and 100 µL of PBS was added. CellTiter Glo^®^ reagent (Promega, Madison, WI, USA) was prepared according to manufacturer’s instructions, and 100 µL was added to the wells. The plates were shaken for two minutes and equilibrated at room temperature for 10 min prior to measuring the luminescence using Varioskan LUX multimode plate reader (Thermo Scientific, Vantaa, Finland). Inhibition of cell viability was calculated in relation to the untreated cells. 

### 3.9. Statistical Analysis

Statistical parameters signal-to-noise (S/N), signal-to-background (S/B) and Z′ values were calculated to monitor the assay performance. Unpaired *t*-test with Welch’s correction was used to assess the significance using GraphPad Prism v. 7.0 software (GraphPad software Inc., San Diego, CA, USA). *p* < 0.05 was considered as statistically significant. During the primary screening, compounds were tested as single well compounds, while in follow-up studies, compounds were tested in three technical replicates, three times. The IC_50_ values were calculated from 15 concentration points by non-linear regression analysis (sigmoidal dose-response fitting with variable slope) using GraphPad Prism. 

## 4. Conclusions

The study demonstrates feasibility of the previously optimized screening platform as an efficient tool for the identification of naturally inspired QSIs against Gram-negative bacteria. It identified several structurally different, highly active QSIs against *C. violaceum*. In addition to violacein inhibition, the follow-up studies revealed that the selected lead compounds interfered with other QS-mediated functions, namely, biofilm maturation and motility of other Gram-negative species (*P. aeruginosa* and *E. coli*) without inhibiting the growth. The selected leads reduced the transition from microcolonies to fully formed biofilms to a varying extent in *E. coli* and *P. aeruginosa*, and significantly inhibited swimming and swarming motilities of *P. aeruginosa*. These findings indicate broad-spectrum anti-QS activity. The leads were able to affect biofilm lifecycle at different stages of development, which further emphasizes the potential of the leads to be used as adjunctive agents with conventional antibiotics. Further studies with these compounds could include determination of the effects on biofilm dispersal and virulence factors production, as well as combinatory testing with conventional antibiotics. Moreover, these results suggest that the lead compounds may serve as inspiring scaffolds for further optimization towards more efficient, non-toxic QSIs to control Gram-negative bacteria. 

## Figures and Tables

**Table 1:** Inhibition-% of violacein production by the identified highly active compounds in the primary screening.

Compound	Compound ID	ATCC 31532	CV026	Class
**144**	ST012391	94.7	97.2	Nucleoside analogue
**339**	ST024776	95.6	94.7	N/A, a mixture of two compounds *
**341**	ST024784	93.4	95.2	Flavonoid (isoflavonoid)
**575**	ST045414	94.1	94.3	Flavonoid (flavonol)
**916**	ST069294	101.9	95.7	Flavonoid (flavone)
**1698**	ST077117	91.3	96.1	Lignan
**2117**	ST083092	101.8	101.9	Flavonoid (flavone)
**2307**	ST088527	98.8	98.6	Alkaloid
**2896**	ST079962	94.5	91.2	Flavonoid (flavone)

* Penicillin G, salt with 4-[[2-(diethylamino)ethoxy]methyl]aniline; N/A = not applicable.

**Table 2:** Inhibition-% of violacein production by the highly active compounds at 40 µM and IC_50_ values of the leads with 95% confidence intervals. Results are reported as mean ± standard deviation (SD) of three independent tests. N/A = not applicable, N.A. = not analyzed, Que = quercetin. The IUPAC names of the compounds are listed in Table S4.

Compound	Class	Inhibition-% ^1^	IC_50_ (µM)	95% Confidence Intervals
**144**	Nucleoside analogue	0 ± 5.2	N.A.	
**339**	N/A	3.7 ± 1.8	N.A.	
**341**	Flavonoid (isoflavonoid)	26.9 ± 7.7	N.A.	
**575**	Flavonoid (flavonol)	60.9 ± 4.1	N.A.	
**916**	Flavonoid (flavone)	35.8 ± 1.1	N.A.	
**1698**	Lignan	2.6 ± 4.6	N.A.	
**2117**	Flavonoid (flavone)	99.7 ± 1.0	9.6	7.9–11.8
**2307**	Alkaloid	76.1 ± 3.3	N.A.	
**2896**	Flavonoid (flavone)	83.5 ± 1.4	13.9	8.5–22.5
Que	Flavonoid (flavonol)	91.8 ± 2.3	3.1	2.1–4.5

^1^ Tested in *C. violaceum* ATCC 31532.

**Table 3:** Inhibitory activity of the leads (at 400 µM) on the transition from microcolonies to fully formed biofilms as quantified using crystal violet staining assay. Results are reported as mean ± SD of three independent experiments.

Strain	2117	2896
*P. aeruginosa* PAO1	42.3 ± 12.4	11.6 ± 11.1
*P. aeruginosa* ATCC 9027	27.2 ± 6.0	22.5 ± 3.2
*P. aeruginosa* ATCC 15422	11.2 ± 1.8	28.1 ± 15.1
*E. coli* K-12	51.3 ± 6.9	14.3 ± 10.1

**Table 4:** Inhibition-% of cell viability after 24 h exposure to the lead compounds. Results are reported as mean ± SD, *n* = 3. N.I. = no inhibition. Cytotoxicity was tested in human lung (HL) epithelial cells and mouse monocyte macrophage (RAW 264.7) cells.

Compound	Concentration	HL	RAW 264.7
**2117**	100 µM	11.2 ± 4.9	97.9 ± 0.5
**2896**		N.I.	N.I.
**2117**	40 µM	N.I.	N.I.
**2896**		N.I.	N.I.
**2117**	10 µM	N.I.	N.I.
**2896**		N.I.	N.I.
Usnic acid	200 µM	90.0 ± 4.7	98.5 ± 0.6

## Supplementary Materials

Supplementary materials can be found at http://www.mdpi.com/1422-0067/19/5/1346/s1.

## Abbreviations

QS	Quorum sensing
QSI	Quorum sensing inhibitor
AHL	*N*-acyl-l-homoserine lactone
AI	Autoinducer
HSL	Homoserine lactone
QQ	Quorum quencher
LBY	Luria–Bertani broth supplemented with yeast extract
QUE	Quercetin
