# Supplementary material for: Screening of Natural Product Derivatives Identifies Two Structurally Related Flavonoids as Potent Quorum Sensing Inhibitors against Gram-Negative Bacteria
Source: Int J Mol Sci. 2018 May 3;19(5):1346. doi: 10.3390/ijms19051346 (PMC5983823; doi:10.3390/ijms19051346)

Supplementary table 1. Compound codes, IUPAC names, inhibition percentages and structures of the quorum quenchers (QQs) identified in the primary screening. Structures are drawn using ACD/ChemSketch software.

Compound	Compound code (supplier) and IUPAC name	Inhibition-% against *C. violaceum* CV026	Structure
**712**	**ST055915**<br>2-{1-[(4-chlorophenyl)carbonyl]-5-methoxy-2-methylindol-3-yl}-N-(3-piperidylpropyl)acetamide	94.3	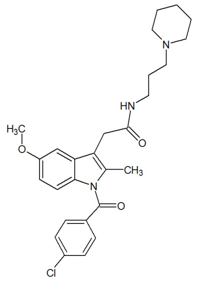
**1016**	**ST070387**<br>methyl 5-cyano-2-methyl-6-sulfanyl-4-(3,4,5-trimethoxyphenyl)-1,4-dihydropyridine-3-carboxylate, morpholine	97.5	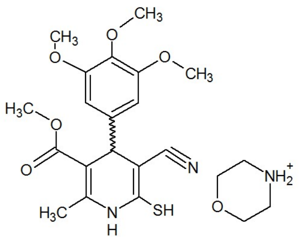
**1214**	**ST072415**<br>2-[(2,5-dimethyl(3-furyl))carbonylamino]-3-phenylpropanoic acid	103.0	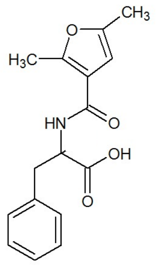
**1352**	**ST074558**<br>3-(4-dichlorophenyl)-4,7-dimethylchromen-2-one	99.6	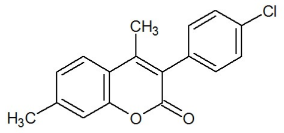

**1406**	**ST074754**<br>2-[4-(N,N-diethylcarbamoyl)-2-oxohydroquinolyl]-N-(2,4,6-trimethylphenyl)acetamide	93.3	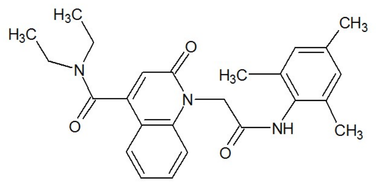
**1508**	**ST075134**<br>N-(3-ethyl-4-oxo(3-hydroquinazolin-7-yl))(3,4,5-trimethoxyphenyl)carboxamide	93.4	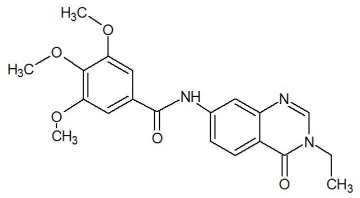
**1852**	**ST078265**<br>6-ethoxy-4-(piperazinylmethyl)chromen-2-one	95.2	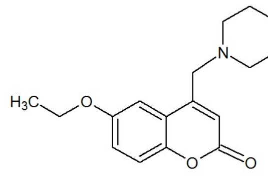
**1905**	**ST078576**<br>N-(3-chloro-4-fluorophenyl)-2-[6-oxo-9-(oxolan-2-ylmethyl)hydropurin-8-ylthio] acetamide	106.7	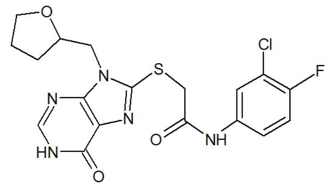
**2372**	**ST092296**<br>(2E)-1-(4-hydroxy-2-methylphenyl)-3-(4-methoxyphenyl)prop-2-en-1-one	91.1	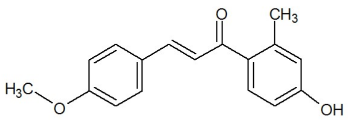
**2584**	**ST095355**<br>2-(2-piperidyl)acetamide	106.4	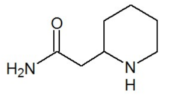

**2666**	**ST007136**<br>4-ethoxy-1-(1,2,3,4-tetrahydrobeta-carbolinyl)benzene	90.8	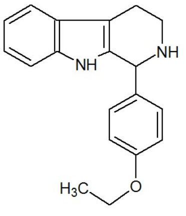
**2712**	**ST034304**<br>2-(trichloromethyl)chromen-4-one	106.2	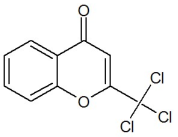
**2909**	**ST081483**<br>3-(2-methoxyphenyl)-4-oxo-2-(trifluoromethyl)chromen-7-yl 2-fluorobenzoate	99.9	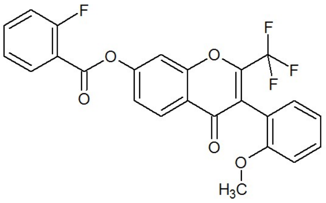
**2916**	**ST081723**<br>3-(3-fluorophenyl)-N-[2-hydroxy-1-(hydroxymethyl)-2-phenylethyl]propanamide	98.3	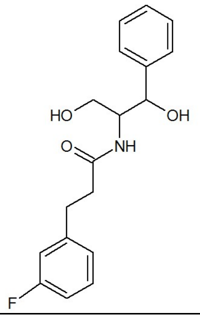

Supplementary table 2. Primary screening of NDL-3040 library.

Cpd	Company ID	Inhibition of violacein (%)		Inhibition of viability (%)	
		ATCC	CV026	ATCC	CV026
1	ST000055				
2	ST000291	19.0			
3	ST001314	41.0	41.3		
4	ST001350				
5	ST001351	8.1	21.9		
6	ST001454		13.0	51.3	
7	ST001455	25.4	8.2		
8	ST001456	4.9			43.9
9	ST001458		7.6		47.9
10	ST001460	17.2			51.2
11	ST001462				
12	ST001503				
13	ST001557				
14	ST001560	99.1	35.3	40.1	48.6
15	ST001561				
16	ST001577			60.9	
17	ST001593				65.5
18	ST001626	8.3	83.7		
19	ST001635				
20	ST001691		31.6		
21	ST001732	7.3	31.4		
22	ST001875	14.9	5.4		
23	ST001922			48.0	
24	ST001934	10.7		60.2	
25	ST001941	33.3	6.8		
26	ST002011	66.0	57.6		
27	ST002046		22.1		
28	ST002048				
29	ST002049				
30	ST002075	12.1	27.4		
31	ST002086				
32	ST002217	2.4	1.1	44.6	42.6
33	ST002232	48.7	2.8		47.8
34	ST002262		33.9		
35	ST002321		16.3		58.9
36	ST002489		43.2		
37	ST002695				
38	ST002729				51.1
39	ST002734	80.4	51.8	46.2	
40	ST002787	18.7	15.7		
41	ST002793		39.0		64.6
42	ST002794		27.3		44.2
43	ST002795				
44	ST002796	3.9	34.5		
45	ST002797		5.4		
46	ST002799				

47	ST002870		26.5		
48	ST002875	6.8	21.7		
49	ST002905	86.8	96.9		
50	ST002906	69.9	87.6		
51	ST003334	32.6	54.3		
52	ST003848		1.9		
53	ST004334	88.2	77.6		
54	ST004335	95.9	79.1		
55	ST004388				
56	ST004882				57.3
57	ST005054	6.3			
58	ST005055				
59	ST005056				
60	ST005058	19.5	35.1		
61	ST005059				
62	ST005063		15.6		
63	ST005069		4.4		
64	ST005078	8.1			
65	ST005080		17.0		47.5
66	ST005085		40.9		
67	ST005086				
68	ST005087		14.2		51.2
69	ST005092				
70	ST005106		9.2		
71	ST005109	0.3			
72	ST005114	28.7	71.8		
73	ST005130	85.9	86.3		47.3
74	ST005132	92.3	26.2		42.5
75	ST005133	91.2	16.4		
76	ST005134	77.1	32.0		
77	ST005135	57.3	29.3		
78	ST005136	88.1	50.8		
79	ST005138	67.9	28.4		
80	ST005179		19.6		
81	ST005183	63.4	49.9	46.0	52.9
82	ST005185	54.8	27.1	51.5	
83	ST005186	59.4	56.4	57.5	
84	ST005190	56.7	55.1	52.8	
85	ST005197	49.6	46.9		
86	ST005199	53.8	58.3		
87	ST005200	57.8	62.3	47.7	43.7
88	ST005201	64.4	45.3	74.0	43.9
89	ST005206	46.8	24.5	59.8	
90	ST005208	54.6	41.0		
91	ST005211	51.8	20.5	57.1	
92	ST005213	57.7	43.5	49.6	
93	ST005216	67.3	11.4		43.5
94	ST005222	52.6	10.2	40.8	
95	ST005223	52.8	16.9	46.1	52.3
96	ST005635	65.5	42.5	73.2	51.3
97	ST005846	48.5	30.5	52.1	

98	ST005872	52.8	41.3	73.4	
99	ST005874	57.5	36.7	58.5	
100	ST005893	53.8	36.0	64.3	
101	ST005930	89.0	55.0		
102	ST006037	55.6	10.6	60.6	50.4
103	ST006587	55.0	64.2		57.4
104	ST006593	58.2	44.6	53.0	
105	ST007000	53.6	17.9	46.6	55.0
106	ST007035	99.0	99.9	99.3	99.5
107	ST007286	58.9	32.3		60.1
108	ST007299	61.2	56.8		
109	ST007922	69.9	39.6		
110	ST007974	60.1	30.3		50.0
111	ST008364	60.5	34.1		
112	ST008365	66.0	56.8		
113	ST008598	54.0	15.0		61.0
114	ST008655	59.2	28.0		46.0
115	ST008666	63.0	35.4	58.9	45.9
116	ST008985	55.0	37.0	64.4	
117	ST008990	68.6	49.5		
118	ST009494	82.6	79.2	62.1	73.2
119	ST009820	87.0	88.7		
120	ST009866	64.7	35.0		
121	ST009869	65.4	37.4	51.8	59.1
122	ST009872	46.4	25.2		
123	ST010134	46.0	26.0		
124	ST010178	49.9	56.6		
125	ST010196	61.7	38.2		
126	ST010199	48.9	30.1		46.2
127	ST010204	40.7	7.2	45.0	51.4
128	ST010548	70.4	53.6		
129	ST010639	46.2	44.1	58.6	
130	ST010647	53.9	69.8	72.7	
131	ST010648	59.5	34.9	54.6	
132	ST011269	50.3	30.9	74.3	
133	ST011381	57.4	49.3	69.0	
134	ST011566	66.1	43.1		
135	ST011915	44.3	4.5	67.4	
136	ST011916	55.3	22.8	101.5	
137	ST011966	42.5	6.7		51.9
138	ST011968	36.7	26.1		57.5
139	ST011982	45.5	18.9		57.1
140	ST012102	54.9	49.2	52.2	
141	ST012322	50.8	46.8	45.4	
142	ST012373	46.3	15.2	56.1	43.5
143	ST012388	51.8	11.4		49.3
144	ST012391	94.7	97.2		
145	ST012395	59.7			51.5
146	ST012416	43.9	22.1		60.6
147	ST012454	46.9	21.6		
148	ST012706	51.7	57.2		

149	ST012707	46.8	50.8		
150	ST012714	58.6	20.4		53.9
151	ST012799	60.3	22.5		49.1
152	ST012804	71.6	96.9		
153	ST012873	40.0	8.9		67.9
154	ST012882	76.6	78.8		56.5
155	ST012883	51.2			42.9
156	ST012885	92.3	68.5	46.7	75.7
157	ST012886	96.7	56.1		61.6
158	ST012887	87.4	64.2		54.9
159	ST012891	87.1	83.6		52.8
160	ST012892	97.4	90.2	47.2	68.8
161	ST012900	41.9	36.8		56.6
162	ST012915	26.7	45.7		
163	ST012935	2.8		40.4	45.5
164	ST012939	98.6	95.4		98.5
165	ST012940	96.1	82.0	83.5	101.3
166	ST012949	54.0	55.0		
167	ST012956	92.7	69.1		
168	ST012957	90.1	84.6		
169	ST012960	97.0	96.9	98.3	98.1
170	ST013021	54.0	83.7		
171	ST013038	35.5	78.7		
172	ST013043		70.3		
173	ST013044	40.4			
174	ST013114		47.9		
175	ST013134		53.5		
176	ST013141		34.7		
177	ST013145	32.5	67.6		
178	ST013165		29.8		
179	ST013170	14.3	59.8		
180	ST013172		61.2		
181	ST013175		41.2		
182	ST013195	74.8	95.1		
183	ST013222	62.7	74.0		
184	ST013224	11.8	47.3		
185	ST013226	22.8	65.8		
186	ST013233		54.1		
187	ST013299		52.6		
188	ST013307	19.0	46.2		
189	ST013313	8.4	44.5		
190	ST013335	52.1	79.8		
191	ST013336	9.3	65.2		
192	ST013355		65.2		
193	ST013357		19.3		
194	ST013362		31.6		
195	ST013440		38.6	40.1	
196	ST013845	9.3	40.1		
197	ST013859		52.0		
198	ST013863	6.1	80.2		
199	ST013865		89.2	52.7	

200	ST013874	13.9	38.2	41.7	
201	ST013875	20.6	48.5		
202	ST013885		33.6		
203	ST014085		51.9		
204	ST014287		54.3		
205	ST014351	12.0	55.1		
206	ST014439		72.9		
207	ST014458		20.1		
208	ST014701	32.3	59.1		
209	ST014848	46.6	67.6		
210	ST014917	39.5	61.7		
211	ST015000	44.1	37.3		
212	ST015003		55.8		
213	ST015022		59.1		
214	ST015025		55.9		
215	ST015028		41.0	41.8	
216	ST015061	3.5	59.5		
217	ST015079	25.1	74.4	58.1	98.2
218	ST015082	16.7	66.0		
219	ST015143	59.7	62.2		
220	ST015200		62.1		
221	ST015213	52.4	68.6		
222	ST015228	21.4	61.8		
223	ST015229	12.2	54.3		
224	ST016345	37.6	56.7		
225	ST016598	89.9	98.6		
226	ST016609	4.9	2.8		
227	ST016611	33.9	9.7		
228	ST016912		35.6		
229	ST017012	43.7	56.5		
230	ST017050	30.9			
231	ST017055	9.3	7.9		
232	ST017059	54.3			
233	ST017060	33.7	64.9		
234	ST017091	26.6	65.4		
235	ST017117	43.0	52.3		
236	ST017131		52.6	44.0	
237	ST017132	65.1	76.7		
238	ST017140	47.5	73.4		
239	ST017162	8.4	47.7		
240	ST017243	51.4			
241	ST017282	13.3	11.1		
242	ST017363	36.8	30.1		
243	ST017700	61.2	19.9	65.1	
244	ST018357	8.8	38.0		
245	ST018474	98.4	97.5	87.8	
246	ST018508	74.3	97.7	68.3	
247	ST018530	60.8	0.5	61.0	
248	ST018637	27.3	13.8		43.9
249	ST018723	16.7	4.7		47.9
250	ST018738	7.5	2.1		51.2

251	ST018862	26.4	4.4		
252	ST018944	19.0			
253	ST018956	46.2	35.4		
254	ST019060	37.8	8.7		48.6
255	ST019200	36.4			
256	ST019346	98.4	74.5	66.3	
257	ST019380	10.2	21.2		65.5
258	ST019494		2.3		
259	ST019500		17.1	59.6	
260	ST019600		7.2		
261	ST019653	24.6	27.6		
262	ST019686	48.4	0.5		
263	ST019691	36.6			
264	ST019694	35.3	1.0		
265	ST019695	66.6	20.4		
266	ST019697	43.9			
267	ST019740	18.2			
268	ST019742	15.5			
269	ST019743				
270	ST019771	41.6			
271	ST019809	33.3	12.6		
272	ST019810	62.6	55.3		42.6
273	ST019811	46.9	0.0	47.5	47.8
274	ST019823	41.2	35.9		
275	ST020202	31.0			58.9
276	ST020536		8.6		
277	ST020706		12.2	70.5	
278	ST020714				51.1
279	ST020838	31.6	13.6		
280	ST021016	50.4	35.6		
281	ST021061	47.3	2.4		64.6
282	ST021133	35.2		50.8	44.2
283	ST021156	14.6		53.3	
284	ST021182	48.5	19.1		
285	ST021190	28.1			
286	ST022015	59.6	32.3		
287	ST022034	35.6	45.6	50.3	
288	ST022146	44.1	28.6		
289	ST022345	54.6	2.9		
290	ST022366	16.6			
291	ST022376	31.0	7.0		
292	ST022472	25.5	9.6		
293	ST022487	44.0			
294	ST022497				
295	ST022505	18.0	9.9		
296	ST022512		22.5		
297	ST022518	75.6	87.2		
298	ST022520	89.9	65.2		
299	ST022533	39.4	23.2		
300	ST022660	44.6	27.7		
301	ST022689	65.1	78.9		

302	ST023393	19.0	15.6		
303	ST023394	64.5	19.6		
304	ST023411	31.3	23.0		
305	ST023485	43.4			47.5
306	ST023486	17.7			
307	ST023895	55.2	27.6		
308	ST023898	12.5	3.8		51.2
309	ST023941	27.4	17.6		
310	ST023988	30.3	24.6		
311	ST023990	9.9			
312	ST024009	48.0	55.3		
313	ST024059	48.3			47.3
314	ST024082			52.2	42.5
315	ST024083	36.0			
316	ST024086	58.4	31.8		
317	ST024093	25.8			
318	ST024094	35.3			
319	ST024097	3.8			
320	ST024144	45.7	25.9		
321	ST024291	4.1	75.3		
322	ST024321	66.5	69.7		
323	ST024425		37.0		
324	ST024453	21.8	46.2	53.1	
325	ST024514	3.5	63.0	56.8	
326	ST024526	23.8	87.2		
327	ST024529	11.1	58.2		
328	ST024545		46.6		
329	ST024567	19.6	41.3	60.1	
330	ST024574	12.9			
331	ST024596	27.0	46.7		
332	ST024605	4.8	32.3		
333	ST024616	37.2	54.8		
334	ST024723	14.6	79.0	87.8	
335	ST024725		44.9	68.2	
336	ST024735	20.2	56.4		
337	ST024746		66.2	63.3	46.6
338	ST024767	19.3	57.3	71.9	
339	ST024776	95.6	94.7		
340	ST024782	92.4	95.5	101.6	
341	ST024784	93.4	95.2		
342	ST024793	2.2	57.9		
343	ST024948		58.6		
344	ST024949		64.6		
345	ST024950	38.1	44.7		
346	ST025067	56.1	44.7		
347	ST025090	32.7	52.1		
348	ST025103	3.3	48.8		
349	ST025230	10.9	47.3		
350	ST025236	8.2	48.9		
351	ST025428	23.2	64.1		
352	ST025481	11.0	54.5		

353	ST025534		30.4		
354	ST025539	33.2	74.0		
355	ST025549	28.4	54.2		
356	ST025561	5.2	44.5		
357	ST025581		50.1		
358	ST025591	14.2	70.7		
359	ST025615	1.6	62.9		
360	ST025699	24.4	54.5		
361	ST026111	32.8	68.6		45.2
362	ST026255		55.5		
363	ST026551	1.6	51.9		
364	ST026815	32.1	75.3		
365	ST026921	62.6	74.9		
366	ST026922	86.0	88.2		
367	ST026946		61.3		
368	ST027058	28.1	60.8		
369	ST027168	2.9	54.6	65.0	
370	ST027326	1.0	77.1		
371	ST027385	4.0	62.1		
372	ST027388		58.1		
373	ST027415	26.7	69.1		
374	ST027493	13.0	49.8		
375	ST027509	6.4	54.7		
376	ST027530		62.8		
377	ST027561	41.1	49.0		
378	ST027719		36.2		
379	ST028394	24.6	70.9		
380	ST028455		44.5		
381	ST028624		54.7		
382	ST028626	2.8	39.1		
383	ST028644		51.0		
384	ST028708	39.7	62.4		
385	ST028777		75.2		
386	ST028823		44.5		
387	ST029018	19.1	64.8		
388	ST029234	11.4	73.2		
389	ST029446	13.6	60.5	91.0	
390	ST029463	17.1	71.1		
391	ST029579	35.3	67.6		
392	ST029634	19.6	57.8		
393	ST029675	88.6	98.4	64.7	
394	ST029711		64.9		
395	ST029713	17.2	69.7		
396	ST029810	2.5	63.1		
397	ST029812		71.1	54.2	
398	ST029852		74.4	65.4	
399	ST029862		71.8	65.1	
400	ST029877	47.6	81.9		
401	ST029879	3.4	21.4	47.2	
402	ST029892	17.1	46.0		
403	ST029917	19.7	39.9		

404	ST029993	37.5	53.7		
405	ST030091	29.7	31.7		
406	ST030149	34.9	36.3		
407	ST030406	50.1	48.8		
408	ST030492	64.5	76.5		
409	ST030518	71.7	23.8		
410	ST030589	7.9	13.6		
411	ST030874	34.2	52.5		
412	ST030885	1.9	24.4		
413	ST031151				
414	ST031276	28.5	38.6		
415	ST031278	39.1	7.3		
416	ST031283	25.2	57.9		
417	ST031410	43.9	0.7		
418	ST031841	22.3			
419	ST031858	26.9	32.3		
420	ST032067	96.0	94.6		98.7
421	ST032140	12.9	42.1		
422	ST032169	47.5	7.0		
423	ST032199	52.6	25.6		
424	ST032221	66.3	51.3		
425	ST032227	15.4	31.9		
426	ST032388	11.5	18.9		
427	ST032423	0.1	27.4	43.8	
428	ST032483	9.8	30.2		
429	ST032713	19.2	27.4		
430	ST032792	56.0	31.4		
431	ST033105	88.9	85.4		
432	ST033161	41.9	49.2		
433	ST033261	18.1	19.6		
434	ST033513	22.2			
435	ST033568	1.0	2.6		
436	ST033644	40.8	36.7		
437	ST033653	6.1	20.2		
438	ST034022	21.7	60.7		
439	ST034078	36.0	26.4		
440	ST034244	59.7	61.3		
441	ST034338	34.7	27.1		
442	ST034726				
443	ST034727	15.3	19.4		
444	ST034730	17.6	29.6	52.7	
445	ST034732				
446	ST034733	18.7	14.9		
447	ST034735		27.4		
448	ST035320	83.4	51.5		
449	ST035419	29.2	37.9		
450	ST035454	9.8	33.5		
451	ST035473	12.6	14.9		
452	ST035612	74.2	56.7		
453	ST035691	46.0	37.8		
454	ST035963	31.9	65.0		

455	ST036586	35.1	22.4		
456	ST036601	56.4	43.3		
457	ST036717	24.5	28.2		
458	ST037127	8.6			
459	ST037249	10.5			
460	ST037252	2.9	23.2		
461	ST037435	18.4	43.6		
462	ST037589	12.5	7.2		
463	ST037628	17.9	77.6	58.3	
464	ST037716	46.7	28.0		
465	ST037730	20.5			
466	ST037733	8.9			
467	ST037793	18.2	12.0		
468	ST037807	29.4	78.8	50.6	
469	ST038122	18.5	41.1		
470	ST038325	91.5	52.3		
471	ST038459	27.0	40.8	50.6	
472	ST038469	55.3	60.1		
473	ST038535				
474	ST038608		3.6		
475	ST038639				
476	ST038647		18.9	44.2	
477	ST038694		12.3	47.8	
478	ST038696		50.8		
479	ST038702		25.7		47.0
480	ST038741		8.4		
481	ST038762	27.2	31.6		
482	ST038805	10.0	24.8		
483	ST038806				
484	ST038808	5.3			
485	ST038809	31.8	40.0		
486	ST038810	10.7	3.0		
487	ST038811	12.3	36.8		
488	ST038812	34.4	78.1		
489	ST038877	3.6			
490	ST038990	28.7			
491	ST039046	52.6	14.5		
492	ST039051				
493	ST039101	60.6	82.8		
494	ST039113	1.1			
495	ST039129	0.0			
496	ST039132				
497	ST039147		52.0		
498	ST039158		52.0		
499	ST039199		4.3		
500	ST039282		8.0		
501	ST039367		26.5		
502	ST039370		1.7		
503	ST039371		31.1		
504	ST039378		50.2		
505	ST039414		5.1		

506	ST039480		4.6
507	ST039608		25.2
508	ST039659		10.9
509	ST039857	34.0	50.5
510	ST039880	1.3	18.0
511	ST039913		10.8
512	ST040023	13.2	46.0
513	ST040110		6.9
514	ST040237	15.6	24.1
515	ST040658		12.0
516	ST040664	8.3	9.0
517	ST040822		17.0
518	ST040829		22.1
519	ST040847		40.2
520	ST041015	69.1	94.9
521	ST041612		
522	ST041789	10.8	18.2
523	ST041792		34.6
524	ST041796	45.0	55.0
525	ST041880		41.6
526	ST041929		31.0
527	ST041930		36.4
528	ST041931		
529	ST042040		
530	ST042161	46.7	
531	ST092788	24.4	
532	ST042165	43.8	52.3
533	ST042372		
534	ST042674	12.7	
535	ST042763		0.8
536	ST042848	14.5	47.9
537	ST042942		
538	ST042960		
539	ST042961		
540	ST042962		
541	ST042968		
542	ST042970		
543	ST042976		
544	ST042983		0.1
545	ST043059		36.1
546	ST043061	8.2	
547	ST043078		
548	ST043135	18.2	12.3
549	ST043173		21.8
550	ST043180	16.7	13.2
551	ST043224	78.8	91.7
552	ST043226	11.4	33.2
553	ST043227		13.7
554	ST043243	38.9	83.3
555	ST043247	7.9	
556	ST043268	7.6	

557	ST043269	10.1	32.4
558	ST043353		6.4
559	ST043392	20.5	41.4
560	ST043420	55.7	30.2
561	ST043440	8.0	4.2
562	ST043487		
563	ST044056	62.1	21.1
564	ST044494	7.7	23.8
565	ST044551		9.2
566	ST044552		14.1
567	ST044561		17.6
568	ST044630	0.5	56.1
569	ST044974	38.7	54.1
570	ST045060	3.2	
571	ST045065	26.1	0.6
572	ST045273	26.0	55.3
573	ST045276	29.6	13.5
574	ST045374	20.3	5.8
575	ST045414	94.1	94.3
576	ST045488	45.1	
577	ST045538	24.1	
578	ST045600	25.0	4.7
579	ST045687	56.2	33.7
580	ST046338	32.8	30.8
581	ST046798	44.6	23.0
582	ST046829	98.2	57.0
583	ST047749		0.9
584	ST047874		
585	ST048135		
586	ST048140	61.3	35.2
587	ST048234	68.0	
588	ST048250		22.2
589	ST048369	8.6	2.9
590	ST048386		
591	ST048658	26.3	
592	ST048901	19.4	1.9
593	ST049008		
594	ST049022	94.1	57.2
595	ST049045	54.8	
596	ST049081	57.7	44.1
597	ST049112	22.5	
598	ST049114	49.4	
599	ST049115	47.7	29.7
600	ST049116		
601	ST049173		
602	ST049221	37.2	9.4
603	ST049249		
604	ST049489	78.4	72.0
605	ST049552	10.4	19.5
606	ST049566	40.9	
607	ST049567	12.7	

608	ST049753	40.5			
609	ST049800	22.3			
610	ST049813		9.0		
611	ST049846	51.9	51.8		
612	ST049936	92.5	100.2	68.8	
613	ST050032	12.8	2.4		
614	ST050052				
615	ST050054	30.3	1.2		
616	ST050055	29.3	6.8		
617	ST050098	20.3			
618	ST050100		21.8		
619	ST050106	1.7			
620	ST050115	15.1	11.7		
621	ST050140	59.1	8.3		
622	ST050151	44.4			
623	ST050180	24.2			
624	ST050633	46.3			
625	ST050731	28.3	12.6		
626	ST050780	23.6	31.9		
627	ST051184	44.0			
628	ST051247	67.5	51.4		
629	ST051349	66.4	44.0		
630	ST051365	33.4	2.4		
631	ST051369				
632	ST051546	12.2	18.9		
633	ST051579	25.3			
634	ST051582	13.4			
635	ST051585	20.0			
636	ST051671	73.9			
637	ST051889	34.5			
638	ST052109				
639	ST052110	29.6	12.6		
640	ST052113				44.8
641	ST052116	27.5	4.9		
642	ST052119	17.6			
643	ST052159	93.6	37.1		
644	ST052168	15.4			
645	ST052217	14.6			
646	ST052224	33.1			
647	ST052275	11.3			
648	ST052326	29.1			
649	ST052329		5.6		
650	ST052428		9.4		
651	ST052456	10.4			
652	ST052463	25.4	15.9		
653	ST052468	43.4	25.8		46.8
654	ST052473		35.7		
655	ST052479	30.6			
656	ST052484	34.2			
657	ST052490	15.3			
658	ST052503	1.4			

659	ST052550	9.3	27.6		
660	ST052552	6.5	7.0		
661	ST052560	20.2		56.7	
662	ST052561	0.0	9.1		
663	ST052562	40.2			
664	ST052637	19.5			
665	ST052638	4.4		43.9	65.8
666	ST052704	9.6	6.1		
667	ST052802				
668	ST052818				
669	ST052856	32.9			
670	ST052954	84.8	81.7		
671	ST053197				
672	ST053431	4.6			
673	ST053448	38.8			
674	ST053494	44.4	22.8		
675	ST053662	4.8			
676	ST053741	65.1	37.0		
677	ST054184	66.8			
678	ST054368	76.3	85.7		
679	ST054396	43.4	6.2		
680	ST054410	24.8	50.2		50.2
681	ST054415				
682	ST054547				
683	ST054635	0.3	3.5		52.9
684	ST054863	50.0	48.0		
685	ST054930		25.4		
686	ST054946				
687	ST055064				
688	ST055065	57.8	34.1		
689	ST055066				
690	ST055114				
691	ST055252	98.4	95.5	70.9	99.6
692	ST055268				50.7
693	ST055353	2.4	9.4		
694	ST055419				50.6
695	ST055620	7.2			
696	ST055621				
697	ST055622				
698	ST055628				
699	ST055632				
700	ST055648	6.5	18.5		
701	ST055809	24.4	25.7		
702	ST055810	52.5	3.3		
703	ST055811				
704	ST055817	46.0	10.4		
705	ST055840	25.5			
706	ST055853	18.7			
707	ST055881	8.9	36.5		
708	ST055882	8.4	20.6		
709	ST055886	28.9	2.8		

710	ST055903	4.8			
711	ST055909	53.3	36.6		
712	ST055915		94.3		
713	ST055924				
714	ST056109	82.7	76.1		
715	ST056192	56.0	45.2		
716	ST056202				
717	ST056228				
718	ST056230				
719	ST056231	51.1	61.0		
720	ST056232	47.3	20.3		
721	ST056236	13.3	11.9		
722	ST056237	9.8	16.0		
723	ST056238	54.3	96.8		
724	ST056239	51.7	88.4		
725	ST056240		26.1		
726	ST056241		23.2		
727	ST056242	73.0	72.0		
728	ST056243	74.4	59.2		
729	ST056244	53.8	75.1		
730	ST056245		0.0		
731	ST056362	17.7	32.0		
732	ST056387				
733	ST056516		5.9		
734	ST056547		31.6		
735	ST056689		6.1		
736	ST056692		30.5		
737	ST056740				
738	ST056759	12.1	22.6		
739	ST056764		38.3		
740	ST056913		11.4		
741	ST056918	96.4	95.1		97.5
742	ST057032	42.8	68.3		
743	ST057039	33.1	47.0		
744	ST057042	87.0	94.6		
745	ST057231	11.2	7.2		
746	ST057245				
747	ST057254	18.8			
748	ST057334		17.4		
749	ST057344	89.1	72.6		
750	ST057345	78.8	44.5		
751	ST057361				
752	ST057448	52.4	44.8		
753	ST057513	30.8	38.9		
754	ST057514	45.7	74.1		
755	ST057538	97.8	96.8	98.0	98.8
756	ST057564	9.2			
757	ST057567	33.9	18.4		
758	ST057591	2.3	16.3		
759	ST057628	22.6	16.7		
760	ST057631	12.5	35.4		

761	ST057632	95.7	95.4	97.7	98.4
762	ST057725				
763	ST057731	64.9			
764	ST057804		44.0		
765	ST057890		31.5		
766	ST058035	7.4	55.4		
767	ST058036	8.7	32.1		
768	ST058064		15.6		
769	ST058074	23.4	18.5		43.3
770	ST058101		7.1		
771	ST058126	66.3	17.5		
772	ST058289		2.0		
773	ST058403		13.4		
774	ST058410	29.3			
775	ST058411		44.8		
776	ST058418				
777	ST058420		13.6		
778	ST058433				
779	ST058439	4.9	14.5		
780	ST058444				
781	ST058452	59.8			
782	ST058664	34.7	32.9		
783	ST058680		30.2		
784	ST058803		35.8		
785	ST058867				
786	ST058908	76.3			
787	ST058909	30.5	38.0		
788	ST059035				
789	ST059073	100.0	93.6	96.9	97.9
790	ST059081		24.1		
791	ST059353		24.3		
792	ST059361	80.6	45.8		
793	ST059419	7.7	32.2		
794	ST059434	52.6			
795	ST059648		1.5		
796	ST059828		64.3		
797	ST060103	31.4	45.2		
798	ST060140		3.7		
799	ST060145	2.8	15.7		
800	ST060182	48.3			
801	ST060191	82.5	43.8		
802	ST060193	88.0	20.2		
803	ST060199	63.1			
804	ST060234	57.2	26.5	42.4	
805	ST060287	89.5	46.7		
806	ST060362	47.0		55.0	
807	ST060391	56.2			
808	ST060472	78.9	37.9		
809	ST060804	91.3	87.0		
810	ST060885	33.5	30.5		
811	ST061746	35.8			

812	ST062376	56.0	57.5		
813	ST063372	45.1			
814	ST063382	52.9		40.1	
815	ST064519	62.6		42.9	
816	ST064520	64.3	60.4		
817	ST064560	52.9			56.9
818	ST064561	61.2	51.4		
819	ST064725	58.9	20.1		
820	ST064726	62.3	19.1		
821	ST065321	70.8	16.5		
822	ST065331	80.2			
823	ST065341	75.8	28.2		
824	ST065355	78.6	16.2		
825	ST065402	75.7		64.7	51.4
826	ST065403	65.1			
827	ST065418	66.8	21.5		
828	ST065428	64.5	61.3	62.1	
829	ST065431	99.0	62.8		
830	ST065439	63.0		59.2	
831	ST065455	65.9		45.2	
832	ST065460	69.9	47.5		
833	ST065467	16.5	56.6	62.5	48.4
834	ST065501	42.4	19.0	66.0	45.4
835	ST065512	34.4	57.1		
836	ST065528	1.6		60.4	48.5
837	ST065529	49.4	27.8	63.6	57.4
838	ST065554			57.9	
839	ST065680	14.0	37.0	44.7	
840	ST065757		57.2	69.2	59.6
841	ST065785	38.7		47.8	
842	ST065792	45.9			
843	ST065826	50.6	2.6		
844	ST065916	64.4	54.3	40.6	
845	ST065924	57.7	41.7		
846	ST065925	47.7	15.4	58.0	
847	ST065983	67.7	59.0	63.5	
848	ST066003	89.4	2.1		
849	ST066157	31.3	11.3		
850	ST066190	65.7		45.8	
851	ST066286	56.6	34.0	55.3	
852	ST066287	53.8	16.2	54.8	
853	ST066288	64.0	72.7	42.1	
854	ST066294	49.0	60.0	65.3	47.3
855	ST066330	81.2	37.6		
856	ST066400	52.9		58.1	54.7
857	ST066425	62.6	10.0		
858	ST066440	33.3	2.5		
859	ST066484	58.3	43.3		
860	ST066505	72.7	28.3		
861	ST066506	77.4	42.4		
862	ST066507	83.9	37.9		

863	ST066681	77.8			
864	ST066694	76.6	30.6	44.8	
865	ST066815	73.9	36.6		
866	ST066817	83.3	1.7		
867	ST066871	39.5			
868	ST066880	59.5			
869	ST066885	66.7	48.8		
870	ST066892	47.8	17.0		
871	ST066902	91.3	51.6		
872	ST066903	76.0	35.1		
873	ST066940	59.7			48.7
874	ST066947	53.3			74.7
875	ST066973	93.7			
876	ST066980	46.7			
877	ST066983	91.4			
878	ST067007	85.4	25.6		
879	ST067113	42.7		87.2	47.3
880	ST067125	99.8	95.0	99.2	99.8
881	ST067128	88.3	10.5		
882	ST067619	83.2	3.6		
883	ST067888	48.0			
884	ST067892	40.0			
885	ST068124	53.1	16.3		
886	ST068192	81.9			
887	ST068197	77.6	83.3		
888	ST068345	84.2	33.0		52.7
889	ST068815	75.8			
890	ST068817	55.0	79.4		
891	ST068828	0.0			40.6
892	ST068835	52.5			
893	ST068840	24.6			42.0
894	ST068845	18.7		41.3	54.6
895	ST068876	71.3	8.1		
896	ST068908	45.9	9.0		49.5
897	ST068909	53.7	4.4		
898	ST068917	85.0	47.6		
899	ST068919	62.0			
900	ST068988	82.8	30.6		
901	ST069019	55.0			
902	ST069027	96.2	55.7		
903	ST069036	48.3			
904	ST069049	63.5			
905	ST069069	29.9			
906	ST069115	52.4			
907	ST069185	82.7	31.9		
908	ST069189	67.0	13.4		
909	ST069212	39.8			
910	ST069229	33.0			
911	ST069268	86.8	28.2		
912	ST069283	56.1			
913	ST069285	47.5			

914	ST069292	79.4	35.9	
915	ST069293	100.3	46.4	
916	ST069294	101.9	95.7	
917	ST069356	68.8	9.1	
918	ST069437	57.6	21.8	
919	ST069782	70.0	3.2	
920	ST069813	50.1		44.9
921	ST069827	25.1	9.2	
922	ST069845	36.4	57.6	
923	ST069851	79.6	9.7	
924	ST069852	35.3	4.0	
925	ST069853	81.7	15.2	
926	ST069862	48.3	2.1	
927	ST069867	65.8		43.6
928	ST069870	0.0		58.4
929	ST069874	38.7	25.4	
930	ST069880	9.5		
931	ST069882	27.7	61.5	
932	ST069883	62.2	7.4	
933	ST069885	52.7		76.5
934	ST069891	51.0		
935	ST069892	73.7		51.8
936	ST069895	63.5		60.1
937	ST069900	57.4	16.2	
938	ST069904	68.1	13.1	
939	ST069909	60.9	29.9	
940	ST069913	54.2	21.5	
941	ST069914	49.0	20.4	
942	ST069919	24.6		
943	ST069920	56.4		
944	ST069921	54.8		
945	ST069925	29.1	7.6	47.1
946	ST069931	88.8	20.1	
947	ST069932	89.0	30.5	
948	ST069933	88.5	13.2	
949	ST069971	79.3	6.5	
950	ST069978	14.6		
951	ST069984	77.5		
952	ST070003	48.8		
953	ST070071	39.5	2.7	
954	ST070081	12.5		44.3
955	ST070082	0.0		
956	ST070083	16.8		
957	ST070085	40.1		
958	ST070086	37.2		
959	ST070111	23.7		
960	ST070114	62.6		60.7
961	ST070116	12.6	53.5	
962	ST070117	68.6	3.8	
963	ST070120	55.4	15.5	
964	ST070122	71.7	2.8	

965	ST070123	60.9	38.3		
966	ST070125	32.5	24.8		
967	ST070136	21.9	53.9		
968	ST070145	57.4	61.5		
969	ST070161	77.6	24.7		
970	ST070174	29.8	19.1		
971	ST070175	30.9	6.9		
972	ST070176	22.2			
973	ST070177	14.1		62.5	40.9
974	ST070178	31.8			
975	ST070179	10.1	15.6		
976	ST070180	49.5	40.7		
977	ST070181	76.5		50.5	
978	ST070182	9.8			
979	ST070183	59.1	32.4		
980	ST070187	71.5	16.0		
981	ST070188	76.2	46.3		
982	ST070191	68.3	12.0		
983	ST070194	49.9	28.3		
984	ST070196	75.3	82.8		
985	ST070199	85.4	21.8		
986	ST070201	83.3	11.8		
987	ST070203	91.0			
988	ST070204	46.1	3.7		
989	ST070206	67.1	45.7		
990	ST070207	87.1	23.8		
991	ST070208	43.1	53.7		
992	ST070231	50.6	30.5		
993	ST070232	81.3	4.1		
994	ST070239	32.6			
995	ST070242	69.8			
996	ST070252	22.6			
997	ST070262	54.7			
998	ST070268	93.3	48.6		
999	ST070269	79.4	8.0		
1000	ST070270	51.3	39.8		
1001	ST070271	69.4	19.1		
1002	ST070272	72.4	5.8		
1003	ST070273	57.6			
1004	ST070277	92.6	14.9	59.4	
1005	ST070281	93.7	66.5		
1006	ST070282	94.0	18.9		
1007	ST070284	45.0	73.3	48.1	
1008	ST070287	65.1	41.4		
1009	ST070290	87.8	6.9		
1010	ST070291	69.1	0.4		
1011	ST070297	77.9	4.8		
1012	ST070330	80.7	1.0		
1013	ST070344	67.7	38.3		
1014	ST070345	61.2			
1015	ST070347	55.4	48.0		

1016	ST070387	34.1	97.5		
1017	ST070396	20.3			
1018	ST070408	88.2	17.1		
1019	ST070416	77.2	11.5		
1020	ST070417	34.6			
1021	ST070418	41.4			
1022	ST070419	92.2	29.9		
1023	ST070420	84.2	34.0	42.9	
1024	ST070421	61.0	33.4		
1025	ST070422	68.2			
1026	ST070438	88.1		55.0	
1027	ST070444	86.6			
1028	ST070455	87.8			
1029	ST070456	30.3			
1030	ST070457	92.0	19.3	54.4	
1031	ST070458	98.0	98.6	97.7	97.6
1032	ST070492	71.5	95.4	53.8	
1033	ST070499	55.3			
1034	ST070506	88.8			67.5
1035	ST070595	87.2			
1036	ST070604	81.5	2.0	51.1	
1037	ST070609	23.9			
1038	ST070619	95.3	25.8	58.9	
1039	ST070626	95.9	95.8	97.7	97.0
1040	ST070645	45.6	95.0	72.5	
1041	ST070657	72.8	66.6		
1042	ST070666	14.1	50.6		
1043	ST070667	39.8	68.5	69.8	
1044	ST070691		14.2	73.0	
1045	ST070696	6.8	74.1	59.5	
1046	ST070700	21.2	11.0		
1047	ST070702	55.4	28.8		
1048	ST070712	41.5	61.6		
1049	ST070720	28.8	38.0		
1050	ST070729	38.3	35.4		
1051	ST070736	65.4	82.0		
1052	ST070744		77.6		
1053	ST070750				
1054	ST070761	28.5	26.9		49.2
1055	ST070805	0.9	30.5		96.3
1056	ST070814	18.0	51.6		
1057	ST070815	43.4	53.3		
1058	ST070816		50.8		
1059	ST070817	47.3	52.4		
1060	ST070818	67.4	50.5		
1061	ST070819	8.5	50.8		
1062	ST070820	59.6	49.8		
1063	ST070821	37.4	40.9		
1064	ST070825	52.9	47.8	41.2	
1065	ST070828	55.7	73.8		
1066	ST070841	34.8	71.5		

1067	ST070844		31.0		
1068	ST070845	64.0	98.0		
1069	ST070846	71.7	64.0		
1070	ST070847	79.5	60.8		
1071	ST070848	84.7	61.7		
1072	ST070849	63.3	60.2		
1073	ST070850	55.2	44.2		
1074	ST070851		36.0		
1075	ST070859		73.5		
1076	ST070860		52.6		
1077	ST070861	56.6	65.4		
1078	ST070865	27.0	52.3		
1079	ST070866	74.9	63.2		
1080	ST070867				70.5
1081	ST070869	75.2	65.7		
1082	ST070879		14.3		
1083	ST070911	13.7	64.2	41.3	
1084	ST070934		17.9	65.9	
1085	ST070940		26.8	59.1	
1086	ST070990	9.5	46.1		
1087	ST071048	1.3	72.5		
1088	ST071058				63.1
1089	ST071081		36.7	57.6	
1090	ST071082		9.3		96.6
1091	ST071099	6.4	55.7		
1092	ST071102	21.1	62.0		
1093	ST071103	23.2	76.8		
1094	ST071104		75.8		
1095	ST071105	19.0	79.5		49.4
1096	ST071106		11.0		
1097	ST071107	26.9	8.8	61.8	
1098	ST071108	58.5	37.2	67.1	
1099	ST071109	63.4	52.0		
1100	ST071112	21.8	31.1	50.4	
1101	ST071124	22.3	85.2		
1102	ST071152		18.2	74.3	
1103	ST071169	92.7	100.1	96.5	97.3
1104	ST071178	7.2	78.6	54.6	
1105	ST071180	90.1	25.4	89.4	45.6
1106	ST071191	11.1	44.5		
1107	ST071243	50.6	56.3		
1108	ST071246		5.8		
1109	ST071248		23.4		
1110	ST071254	9.6	42.8		
1111	ST071258	38.1	74.5		
1112	ST071283	91.3	98.9		98.7
1113	ST071307	22.6	16.6		
1114	ST071326	39.7	42.3		
1115	ST071332	14.9	39.3		
1116	ST071368	54.6	26.2		
1117	ST071372	74.2	68.6		

1118	ST071374	47.8	58.0		
1119	ST071376	21.7	80.3		
1120	ST071381	13.3	73.1		
1121	ST071382	58.9			
1122	ST071609	71.0	79.9		
1123	ST071613	58.7			
1124	ST071618				
1125	ST071631	62.1			
1126	ST071652	49.3	39.1		
1127	ST071653	20.2		55.0	
1128	ST071660	19.4			
1129	ST071661	77.4			
1130	ST071662	23.6	75.8		
1131	ST071672	29.2	16.1		
1132	ST071675	36.8			
1133	ST071687	3.4		50.8	
1134	ST071698			64.2	
1135	ST071704	9.3			
1136	ST071705	4.1	50.1	52.2	
1137	ST071761	94.8	62.7	50.1	
1138	ST071764		24.6	51.0	
1139	ST071773	46.2			
1140	ST071804	59.5			
1141	ST071806	40.5			
1142	ST071809		67.8	104.5	
1143	ST071810		18.0	65.3	
1144	ST071811	35.3	20.1	66.4	
1145	ST071812	32.2	33.3	53.6	
1146	ST071813	68.2	57.9		
1147	ST071814	34.5	34.2		
1148	ST071815	62.7	33.7		
1149	ST071816		54.7	66.7	62.8
1150	ST071817			95.8	
1151	ST071819	51.8	59.1		
1152	ST071823	21.5	48.3		
1153	ST071826	72.1	67.7		
1154	ST071827		81.1		
1155	ST071837			66.1	
1156	ST071838	19.6	73.8	50.5	
1157	ST071842		14.4		
1158	ST071843	47.0			
1159	ST071846	62.5	81.9		
1160	ST071857	28.3	60.8		
1161	ST071861		36.5	52.7	
1162	ST071866	80.9			
1163	ST071868			73.8	79.6
1164	ST071869	57.7			
1165	ST071872	44.3	5.2		
1166	ST071873	1.6	30.7		
1167	ST071874		26.2	50.3	
1168	ST071934	30.1			

1169	ST071936				
1170	ST071942				
1171	ST071943				
1172	ST071963	28.1			
1173	ST071988				
1174	ST072000	35.0	40.9		
1175	ST072028	5.1		50.9	
1176	ST072050			46.7	
1177	ST072062	41.3			
1178	ST072068	56.5			
1179	ST072086	15.3			
1180	ST072087	21.4			
1181	ST072088	25.6	42.2		
1182	ST072120	25.1			
1183	ST072189	6.3			
1184	ST072200	94.0	87.9	99.4	98.5
1185	ST072213				
1186	ST072236	67.7			
1187	ST072244	42.6	42.5		
1188	ST072245	33.1	41.2		
1189	ST072246	66.9	9.3		
1190	ST072247				
1191	ST072248	15.2			
1192	ST072283	62.4	58.0	59.4	
1193	ST072284			48.0	
1194	ST072285	58.0	28.5	51.8	
1195	ST072286	72.4		49.8	
1196	ST072290	55.9	70.3	50.0	
1197	ST072291	14.7	47.2		
1198	ST072292	49.1	44.3	74.2	
1199	ST072299			71.5	
1200	ST072300				
1201	ST072303		2.9		
1202	ST072305				
1203	ST072306		15.1		
1204	ST072319	25.0	37.0		
1205	ST072320	30.5	47.9		
1206	ST072321	31.4	41.2		
1207	ST072322		9.9		
1208	ST072326	3.6	65.0		
1209	ST072327		33.1		
1210	ST072383		43.5		
1211	ST072385		41.8		
1212	ST072400		57.5		
1213	ST072414		39.9		
1214	ST072415		103.0		
1215	ST072436		31.1		
1216	ST072460		40.7		
1217	ST072533		28.8		40.4
1218	ST072573		25.5		47.4
1219	ST072956		32.4		43.8

1220	ST072960		38.9		45.0
1221	ST072968		42.5		
1222	ST072975		28.6		
1223	ST072980		68.2		
1224	ST072991		62.8		
1225	ST072994		48.9		
1226	ST072995		41.7		
1227	ST073000		33.3	62.3	
1228	ST073011		35.1		
1229	ST073018		42.3		
1230	ST073028		44.9		
1231	ST073050		51.0		
1232	ST073057		46.7		
1233	ST073059				43.9
1234	ST073066				
1235	ST073073		6.9		
1236	ST073151		30.2		
1237	ST073152		12.0		
1238	ST073153	19.8	0.9		
1239	ST073157				
1240	ST073164		3.4		
1241	ST073165		24.5		
1242	ST073166		35.1		
1243	ST073173		52.6		
1244	ST073174		29.5		
1245	ST073175		15.9		
1246	ST073206		14.1		
1247	ST073219		29.4		
1248	ST073282		31.3		
1249	ST073363		51.6	58.4	
1250	ST073382				
1251	ST073733		26.2		
1252	ST073740		24.5		
1253	ST073741		31.9	43.4	
1254	ST073742		30.2	52.8	
1255	ST073783		32.9		
1256	ST073815		22.4	56.2	
1257	ST073843		16.4		
1258	ST073918		17.2		
1259	ST073946		31.0		
1260	ST073954		49.4		
1261	ST074022		58.2	48.5	49.8
1262	ST074026		39.3		
1263	ST074031		37.9		51.7
1264	ST074036				
1265	ST074266		31.2		42.7
1266	ST074268		64.3		
1267	ST074270	40.7	72.3		
1268	ST074273		30.1	42.4	
1269	ST074282		48.9		44.8
1270	ST074284		37.4		

1271	ST074298		51.2	41.1	
1272	ST074299	27.4			
1273	ST074300	51.1	62.3		96.6
1274	ST074303				
1275	ST074304		32.7		
1276	ST074305				
1277	ST074306		16.0		
1278	ST074307		24.6		
1279	ST074308		50.3		
1280	ST074309		57.7		
1281	ST074310				
1282	ST074311	1.1			
1283	ST074312				
1284	ST074313	24.0			
1285	ST074314	16.7	0.5		
1286	ST074315				
1287	ST074316	13.9	37.2		
1288	ST074317		26.3	63.1	
1289	ST074318	87.5			
1290	ST074344				
1291	ST074345				
1292	ST074346				
1293	ST074347				
1294	ST074348		28.3		
1295	ST074349		17.4		
1296	ST074350		36.0		
1297	ST074351	99.2			
1298	ST074370	1.2			
1299	ST074375				
1300	ST074376		31.4		
1301	ST074377	33.8	21.9		
1302	ST074378		4.2		
1303	ST074379		15.4		
1304	ST074380	6.7	44.7		
1305	ST074381		20.7		
1306	ST074382		14.7		
1307	ST074383		51.0		
1308	ST074384	30.7	45.5		
1309	ST074385		40.3		
1310	ST074386	7.1	46.8		
1311	ST074387	23.8	29.1		
1312	ST074388		40.3		
1313	ST074390		30.0	56.6	
1314	ST074391	39.5		55.0	
1315	ST074392		55.9		
1316	ST074393		29.2		
1317	ST074394		36.0		
1318	ST074395		45.9		
1319	ST074396		38.6		
1320	ST074397		39.0	51.1	
1321	ST074398		34.0		

1322	ST074399		38.2		
1323	ST074400		26.9		
1324	ST074401		35.9		
1325	ST074402		38.1		
1326	ST074403		28.4		
1327	ST074404		39.1		
1328	ST074405		62.7		
1329	ST074406		17.2		
1330	ST074408	23.9	52.8		
1331	ST074412	50.2	47.4	44.9	50.2
1332	ST074429		49.3		
1333	ST074438		53.7		
1334	ST074446		47.5		
1335	ST074454		35.0		
1336	ST074455		57.4		
1337	ST074456		36.1		
1338	ST074466	19.6	70.8		
1339	ST074488		56.6		
1340	ST074489		51.4		
1341	ST074502			48.5	
1342	ST074508		61.5		
1343	ST074515		53.3		
1344	ST074520	29.4	50.9	114.8	
1345	ST074526		71.5		
1346	ST074527		36.1		
1347	ST074528		49.7		
1348	ST074532		44.6		
1349	ST074545		46.2		
1350	ST074554			57.8	
1351	ST074557		26.6		
1352	ST074558	18.8	99.6		
1353	ST074559		35.8		
1354	ST074660		1.5		
1355	ST074701		14.1		
1356	ST074702		36.5		
1357	ST074705		47.6		
1358	ST074706		34.9		
1359	ST074707	20.7	32.9	42.4	
1360	ST074708		39.7	54.4	
1361	ST074709	25.2	20.7	60.2	
1362	ST074710		24.1		
1363	ST074711		58.1		
1364	ST074712	18.3	48.1	70.3	54.6
1365	ST074713	6.4	29.2	71.6	
1366	ST074714	72.4	12.6	49.4	
1367	ST074715	87.9	56.1		
1368	ST074716	24.3	60.8	44.2	
1369	ST074717	25.9	26.8	65.4	46.1
1370	ST074718		33.9		
1371	ST074719		54.4		
1372	ST074720	27.7	57.9		

1373	ST074721	53.2	39.8		
1374	ST074722	55.0	47.2		
1375	ST074723	31.0	63.7		
1376	ST074724	31.5	62.4		
1377	ST074725	42.9	63.2		
1378	ST074726	6.3	22.5	56.5	75.6
1379	ST074727	68.5	22.8	41.3	53.3
1380	ST074728	35.5			56.8
1381	ST074729	53.8	1.5		
1382	ST074730	44.9	39.4		
1383	ST074731	21.5	23.0		
1384	ST074732	87.7	91.9		
1385	ST074733	72.7	92.8		
1386	ST074734	38.2	81.3		
1387	ST074735		26.7		45.2
1388	ST074736	30.6			
1389	ST074737	35.9	27.1		
1390	ST074738	36.9	48.7		
1391	ST074739	29.0	59.2		
1392	ST074740	48.6	55.4		
1393	ST074741		53.5		
1394	ST074742	31.7	54.6		40.3
1395	ST074743	53.8	39.1		
1396	ST074744		27.9		70.3
1397	ST074745		89.2		52.7
1398	ST074746	35.8	48.0		
1399	ST074747	38.1	26.8		
1400	ST074748	64.3	16.3		
1401	ST074749	67.7	34.5		
1402	ST074750	30.0	2.3		59.3
1403	ST074751	80.1	28.0		70.9
1404	ST074752	17.1	26.5		55.4
1405	ST074753	77.0	3.5		
1406	ST074754	40.0	93.3		
1407	ST074755	40.1			
1408	ST074756	2.6	16.6		
1409	ST074757	62.3	27.2		
1410	ST074758	23.1	21.7	49.0	62.2
1411	ST074759	48.3	30.6		
1412	ST074760	25.0		51.6	65.4
1413	ST074761	36.5	10.7		
1414	ST074762	33.0	21.8		
1415	ST074763	39.3	61.8		
1416	ST074764	63.8	59.0		
1417	ST074765	42.9	27.7	55.7	
1418	ST074766	70.9			
1419	ST074767	61.4	38.5	59.9	42.0
1420	ST074768	30.5	25.1		
1421	ST074769	53.2	55.0	53.3	
1422	ST074770	33.9	0.0		
1423	ST074771	27.4			

1424	ST074772	22.8			46.3
1425	ST074773	60.0	50.4		46.9
1426	ST074774	59.4	59.3		
1427	ST074775	30.4	58.3	64.3	52.2
1428	ST074776	17.7	54.0		46.0
1429	ST074777	31.1	58.6		
1430	ST074778	35.5	60.0	51.7	56.2
1431	ST074779	21.5	58.3	54.5	56.0
1432	ST074780	66.1	30.8		40.7
1433	ST074781	3.2	61.1	64.2	58.8
1434	ST074782	23.8	30.6	59.2	69.7
1435	ST074783	41.8	57.2	58.5	59.4
1436	ST074784	40.1	29.9		
1437	ST074785	49.0	30.9		
1438	ST074786	57.7	59.7		
1439	ST074787	53.8	58.0		56.6
1440	ST074788	54.6	62.2		
1441	ST074789				58.6
1442	ST074790		5.3		
1443	ST074791				
1444	ST074792				
1445	ST074793	7.7			
1446	ST074794	10.5			
1447	ST074795	11.2			
1448	ST074796	31.6			
1449	ST074797				
1450	ST074798				
1451	ST074799	14.3			
1452	ST074800	20.9			
1453	ST074801	7.4			
1454	ST074802	42.4			
1455	ST074803	74.0	17.5		
1456	ST074804	17.4			
1457	ST074805	48.2	18.8		
1458	ST074806	65.0	21.2	57.0	57.3
1459	ST074807	5.9			
1460	ST074808		20.9		
1461	ST074809	17.4	13.7		
1462	ST074810		20.5		
1463	ST074811	43.1		53.1	45.8
1464	ST074812	15.4			
1465	ST074813	19.1	80.4	49.0	
1466	ST074814	64.2	87.7	83.8	66.5
1467	ST074815	83.5	85.5	86.8	38.0
1468	ST074816	0.6	59.5		
1469	ST074817	8.8	87.1	83.3	60.1
1470	ST074818	14.1	4.7		
1471	ST074819	16.8	9.9	73.6	
1472	ST074820	36.2	5.4	52.1	
1473	ST074821	36.8	12.3	77.1	68.0
1474	ST074822	88.4	86.3	65.3	44.5

1475	ST074823	76.9			68.1
1476	ST074824	35.1	4.4		
1477	ST074825	57.4			
1478	ST074826				
1479	ST074827	36.5	36.8		
1480	ST074828	9.7	27.4		
1481	ST074829		32.6		
1482	ST074830	27.3	85.3		45.9
1483	ST074831		20.9		
1484	ST074832	20.3	66.7		
1485	ST074964		0.5		
1486	ST074967				
1487	ST074968		21.7		
1488	ST075011	19.2	10.8		
1489	ST075013				
1490	ST075014	4.2	22.3		
1491	ST075032	2.5	20.4		
1492	ST075035	26.2	53.5		
1493	ST075037	36.9	64.9		
1494	ST075038		49.1		
1495	ST075039	25.5	60.7		
1496	ST075048	47.1	46.6		60.4
1497	ST075058		7.1	50.7	46.6
1498	ST075059	35.3			
1499	ST075062	36.4	82.0		
1500	ST075076	35.2	32.3		
1501	ST075077	37.6	31.1	70.2	45.1
1502	ST075087	72.4	30.9		
1503	ST075088	37.5	4.8		
1504	ST075089	48.8	59.6		
1505	ST075105	37.6	23.7	68.6	44.9
1506	ST075111		30.9	73.0	
1507	ST075112	44.3	35.3		
1508	ST075134		93.4		
1509	ST075135	44.6	18.1		
1510	ST075149	28.3	48.8		
1511	ST075156				
1512	ST075157	92.6			
1513	ST075160	30.0	15.8		
1514	ST075163	34.3	30.3		
1515	ST075171	44.4	28.3		
1516	ST075175	47.8	91.8		
1517	ST075178	55.3		60.2	64.0
1518	ST075199	25.8			58.4
1519	ST075206	60.4		56.5	59.9
1520	ST075231	61.2	2.5		
1521	ST075235		35.5		
1522	ST075254	8.6			
1523	ST075287		22.8		
1524	ST075297	5.2	10.4		53.1
1525	ST075303	20.3	17.1		

1526	ST075304		36.2		
1527	ST075318		6.2		
1528	ST075319	18.5		61.0	68.8
1529	ST075320				
1530	ST075321				
1531	ST075322				
1532	ST075323	24.7	14.5		
1533	ST075328		10.1		
1534	ST075389				
1535	ST075446	88.0	59.4		
1536	ST075549		37.3	120.7	
1537	ST075587	45.1	57.8		41.3
1538	ST075588	8.4	41.0		54.4
1539	ST075640		30.7		39.8
1540	ST075655		49.6		40.1
1541	ST075764		41.1		
1542	ST075797		71.0		
1543	ST075799		45.9		
1544	ST075802	20.2	79.5		
1545	ST075820		28.9		
1546	ST075833		2.5	53.8	
1547	ST075840		50.9		
1548	ST075865	41.3	45.9		
1549	ST075866	30.6	77.6	42.5	
1550	ST075876	51.4	75.4		
1551	ST075879	12.4	44.8	49.5	43.9
1552	ST075882	16.4	19.9	68.3	
1553	ST075892		72.7		
1554	ST075896		8.2		
1555	ST075904	24.7	33.7		
1556	ST075906	95.3	86.5	54.3	
1557	ST076300	2.5	41.6		
1558	ST076301		32.7		
1559	ST076302		49.9		
1560	ST076303	16.9	27.8		
1561	ST076304				
1562	ST076305				
1563	ST076306				
1564	ST076307		63.2		
1565	ST076308		37.6		
1566	ST076309	49.8	41.8	50.1	
1567	ST076310		23.5		
1568	ST076311	27.8	68.2		
1569	ST076312				
1570	ST076314				
1571	ST076315	1.8	63.9		
1572	ST076316	59.8	64.1		
1573	ST076317		49.8		
1574	ST076318		11.1		
1575	ST076319				
1576	ST076320				

1577	ST076321				
1578	ST076322				
1579	ST076323				
1580	ST076324	1.1	62.2		
1581	ST076325		11.6		
1582	ST076326				
1583	ST076327		9.8	52.4	
1584	ST076328	46.5	28.6		
1585	ST076329		13.3		
1586	ST076330		35.1		
1587	ST076331	25.1	75.0		
1588	ST076332		52.8		
1589	ST076333	67.6	67.3		
1590	ST076334	18.4	53.8		
1591	ST076337		42.8		
1592	ST076338		25.4		
1593	ST076339				
1594	ST076340				
1595	ST076341				65.4
1596	ST076342		43.5		
1597	ST076343				
1598	ST076344	3.0	55.4		
1599	ST076345		28.0		
1600	ST076346	22.0	53.3	43.1	
1601	ST076347	40.6			
1602	ST076348		40.8		
1603	ST076350		26.9		
1604	ST076351	10.0	52.5		40.5
1605	ST076352	37.3	31.1		
1606	ST076353	49.0	24.2		
1607	ST076354	18.6	43.8		
1608	ST076355	34.5	12.6		
1609	ST076356		23.5		
1610	ST076357	1.8	4.6		
1611	ST076358		8.5		
1612	ST076359	0.9			44.2
1613	ST076361	18.0	85.8		
1614	ST076362	21.3	34.6		
1615	ST076363	26.7	23.0		
1616	ST076364	19.3	43.0		
1617	ST076365	19.3			
1618	ST076366	8.7	9.1		
1619	ST076367	7.1	38.1		
1620	ST076368		47.5		
1621	ST076370		5.9		
1622	ST076372	17.4	35.5		
1623	ST076373	6.4	25.2		
1624	ST076374	3.0	38.9		
1625	ST076375				
1626	ST076376		21.1		
1627	ST076377		21.3		

1628	ST076378		39.1		
1629	ST076379		5.5		
1630	ST076380		24.1		
1631	ST076381			42.4	
1632	ST076382	43.3	54.0		43.6
1633	ST076384			51.8	
1634	ST076385				46.9
1635	ST076386		29.3		
1636	ST076387		16.1		
1637	ST076388	8.7	24.6		
1638	ST076389		34.2		
1639	ST076390	27.3	42.3		
1640	ST076391		16.3		51.6
1641	ST076392		17.7		
1642	ST076393		3.2	43.1	
1643	ST076394		19.4	44.8	
1644	ST076395		3.0		
1645	ST076396		29.6		45.0
1646	ST076397			41.6	
1647	ST076398	23.5	33.7		51.5
1648	ST076399	3.6	35.8	50.0	51.0
1649	ST076400	47.2	51.7		
1650	ST076401		44.9		
1651	ST076402		15.5		
1652	ST076403	12.8	31.0		
1653	ST076405				53.3
1654	ST076406		17.0		
1655	ST076462		12.3	59.3	43.0
1656	ST076508	23.9	35.7		
1657	ST076514		28.5		
1658	ST076515	15.8	5.0		
1659	ST076521		49.7		
1660	ST076522		2.2		
1661	ST076526	10.8	27.7		
1662	ST076527	18.1	44.3		
1663	ST076533	18.4	14.3		
1664	ST076534	7.1	55.1		
1665	ST076538		21.8		
1666	ST076545				
1667	ST076550	2.2	40.5		
1668	ST076613	24.1	42.0		
1669	ST076724		48.9		
1670	ST076727		25.3	44.6	
1671	ST076818		47.8		
1672	ST076837		37.4		
1673	ST076838		35.3		
1674	ST076839		13.7		
1675	ST076848	39.7	45.9		
1676	ST076857	72.7	65.6		
1677	ST076861		21.3		
1678	ST076873		48.4		

1679	ST076874	17.2	32.4		
1680	ST076889	7.5	6.4		
1681	ST076895		60.3		
1682	ST076896		53.4		
1683	ST076904	2.0	28.4		
1684	ST076905	1.9			50.3
1685	ST076906	30.9	64.3		
1686	ST076907		77.6		
1687	ST076913		50.3		
1688	ST076914	41.0	76.0		
1689	ST076915	31.9	63.1		
1690	ST076932	66.3	65.2		
1691	ST076938	9.5	31.5		
1692	ST076942	9.2	56.1		
1693	ST076944	54.3	77.2		
1694	ST076978	32.0	73.4		
1695	ST077036	48.1	70.2		
1696	ST077037	64.4	58.6		
1697	ST077110	87.7	92.5		
1698	ST077117	91.3	96.1		
1699	ST077118	24.3	40.1		
1700	ST077134	39.1	41.0		
1701	ST077135	18.4	43.0		46.6
1702	ST077138	65.2	66.1		55.0
1703	ST077157	60.8	64.6		51.2
1704	ST077158	57.1	57.1		41.4
1705	ST077160	43.2	67.9		
1706	ST077161	58.4	67.4		
1707	ST077163	48.6	59.1		
1708	ST077164	39.7	64.7		
1709	ST077167	61.2	65.5		45.1
1710	ST077168	40.9	62.5		
1711	ST077169	62.7	71.6		
1712	ST077170	31.8	51.1		43.7
1713	ST077171	6.9	71.1		
1714	ST077176	16.4			
1715	ST077195	24.8	72.1		
1716	ST077221	27.3	40.0		
1717	ST077232	90.2	42.2		
1718	ST077236	63.6	58.7		
1719	ST077305	75.5	62.8		
1720	ST077333	21.3	32.2		
1721	ST077334	18.3	79.4	54.2	
1722	ST077335	17.1	63.7		
1723	ST077340	15.8	67.1		
1724	ST077341		51.4	67.0	39.1
1725	ST077344		34.6		
1726	ST077356	71.8	87.2		
1727	ST077382	16.5	47.5		
1728	ST077383	59.2	62.2		
1729	ST077384	31.4	37.4		

1730	ST077385	25.9	23.4		
1731	ST077386	16.9	1.1		80.5
1732	ST077387	36.0	56.7		
1733	ST077388	29.1	57.7		
1734	ST077389	13.4	42.3	58.9	62.6
1735	ST077390	20.4	53.4		
1736	ST077391	46.9	39.9		47.5
1737	ST077392	1.8	46.6		
1738	ST077393	2.9	32.9		
1739	ST077394	20.2	38.8		
1740	ST077395	26.6	44.8		58.1
1741	ST077396	23.8	57.1		
1742	ST077397		53.3		46.6
1743	ST077398	20.3	35.4		53.2
1744	ST077399				76.9
1745	ST077400	9.0	64.6		
1746	ST077401	16.6	21.8		
1747	ST077402		10.2		
1748	ST077403	24.4	43.1		
1749	ST077404	36.0	48.0		
1750	ST077405	21.6	42.0		
1751	ST077406	30.9	30.7		
1752	ST077407	22.6	67.5		
1753	ST077408	15.0	76.0		
1754	ST077409		36.7		
1755	ST077410		22.5		
1756	ST077411	22.5	36.0		
1757	ST077412	3.6	36.5	51.8	
1758	ST077413		41.5		
1759	ST077414	51.0	9.6		53.2
1760	ST077415	41.6	64.4	56.3	48.5
1761	ST077416	54.7	54.0	46.8	
1762	ST077417		25.8	48.6	
1763	ST077418	53.9	59.7		
1764	ST077419	30.1	10.2	49.8	
1765	ST077420	12.1	24.9		
1766	ST077421	35.5	43.5		
1767	ST077422	27.1	60.2		
1768	ST077423	46.0	49.7		
1769	ST077424	37.1	20.7	68.8	
1770	ST077425	24.4	23.5		
1771	ST077426	27.2	19.7	42.5	
1772	ST077427	24.8	47.8		
1773	ST077428	42.2	62.1		
1774	ST077429	20.6	20.6		
1775	ST077430	32.7	16.5		
1776	ST077431	41.4	0.7		
1777	ST077432				
1778	ST077433	36.4	34.8	58.2	
1779	ST077434	34.9		53.9	
1780	ST077435	16.3	11.9		

1781	ST077436	19.1	17.7		
1782	ST077437	0.9	1.7		52.6
1783	ST077438	53.8	43.7		
1784	ST077439	59.1	34.7		
1785	ST077440	10.6	12.1	57.0	58.5
1786	ST077441	16.9	47.6		
1787	ST077442	2.2	24.3	70.5	
1788	ST077443	15.2	34.2	55.4	
1789	ST077444	33.7	41.5		
1790	ST077445	20.7	29.8	56.4	
1791	ST077446	11.9	56.4		
1792	ST077447	28.4	31.3	40.5	
1793	ST077448	1.4	9.9	73.0	68.4
1794	ST077449	19.4	36.6	67.8	
1795	ST077450	17.0	36.3	51.8	
1796	ST077451	26.7	39.3		
1797	ST077452	27.4	41.0	70.2	45.9
1798	ST077453	12.9	24.6	52.8	
1799	ST077454	23.0	44.0	61.3	
1800	ST077455	27.3	40.0	56.5	
1801	ST077456	20.2	30.7		
1802	ST077457	17.4			
1803	ST077458	15.4	28.4		
1804	ST077459	46.2	48.1	42.6	
1805	ST077460	15.3	29.9	40.6	
1806	ST077461	18.7	29.6	42.9	
1807	ST077462	10.2	37.5		
1808	ST077514	54.6	34.6		
1809	ST077581	38.6	43.1	41.9	
1810	ST077614	42.2	69.1	51.7	
1811	ST077732	20.7	48.7	59.6	
1812	ST077733	14.5	37.3	58.0	
1813	ST077755	27.9	54.8	45.7	
1814	ST077771	40.8	66.9	46.7	
1815	ST077906	59.2	61.5	40.0	
1816	ST077912	33.4	4.7		
1817	ST077918	23.6	35.4	46.6	
1818	ST077919			75.9	56.0
1819	ST077923	56.1	72.9		
1820	ST077924	11.1	12.3	61.3	
1821	ST077925	3.7	23.5	69.0	
1822	ST077927	19.4	36.2		
1823	ST077936	14.1	0.1		66.0
1824	ST077942	51.5	45.0	53.6	40.3
1825	ST077946	39.3	47.3		
1826	ST077948	10.3	24.0	73.5	
1827	ST077955	52.0		48.7	53.9
1828	ST077957	26.2	40.1	50.5	
1829	ST077958	24.3	62.1	65.3	
1830	ST077972	36.8	65.6	62.9	
1831	ST078003	28.9	38.7		

1832	ST078016	47.0	36.8	74.9	
1833	ST078022	47.7	72.6	47.9	
1834	ST078024	24.5	45.2		
1835	ST078025	88.1	94.5		99.7
1836	ST078026	0.6	29.0	91.1	
1837	ST078028	49.0	64.3		
1838	ST078029	60.5	68.8		
1839	ST078030	27.1	65.5		
1840	ST078051	39.7	38.7	45.2	
1841	ST078053	2.4	54.8		
1842	ST078054		40.0		
1843	ST078077	4.7	23.8		
1844	ST078078		75.9		
1845	ST078096		40.4		
1846	ST078099		17.7		
1847	ST078109		14.5		
1848	ST078122		46.4		
1849	ST078197		68.6		
1850	ST078242		52.1		
1851	ST078251		64.9	59.6	
1852	ST078265	29.7	95.2		
1853	ST078267		61.0		
1854	ST078268	17.4	86.3		
1855	ST078275		79.6		
1856	ST078278		16.3		
1857	ST078281	99.9	86.1		
1858	ST078287	5.6	66.4		
1859	ST078297	8.0	71.6		
1860	ST078299		54.2		
1861	ST078300		55.2		
1862	ST078301		56.5		
1863	ST078306		77.1		
1864	ST078333		58.8		
1865	ST078336		56.1		
1866	ST078349	11.7	26.0		
1867	ST078350		84.8		
1868	ST078351		83.3		
1869	ST078357		59.1		
1870	ST078458		62.5		
1871	ST078484		59.5		
1872	ST078496				
1873	ST078527		61.1		
1874	ST078528		76.7		
1875	ST078545		86.5		
1876	ST078547		72.4		
1877	ST078548		77.6		38.7
1878	ST078549		77.0		
1879	ST078550		50.4		33.6
1880	ST078551	40.3	66.2		
1881	ST078552		53.4		
1882	ST078553		0.0		

1883	ST078554		25.2		
1884	ST078555		2.3		
1885	ST078556	49.1	80.8		
1886	ST078557		41.2		
1887	ST078558	9.5	7.7		
1888	ST078559		11.8		
1889	ST078560		71.7		
1890	ST078561	29.9	47.5		
1891	ST078562		49.3		
1892	ST078563		15.5		
1893	ST078564		38.7		
1894	ST078565		50.5		
1895	ST078566		26.7		
1896	ST078567		9.3	42.5	51.6
1897	ST078568		50.3		
1898	ST078569				
1899	ST078570		34.0		
1900	ST078571		25.7		
1901	ST078572		33.2		
1902	ST078573		30.1		
1903	ST078574		25.4		
1904	ST078575	7.5			
1905	ST078576	4.7	106.7		
1906	ST078577		41.9		
1907	ST078621		65.7		
1908	ST078653		51.4		
1909	ST078654	1.9	59.2		
1910	ST078655	6.3	78.8		
1911	ST078656		11.2		
1912	ST078657		26.0		50.4
1913	ST078658				
1914	ST078659				
1915	ST078660				
1916	ST078661				
1917	ST078663				
1918	ST078664		7.8		
1919	ST078665				
1920	ST078666				
1921	ST078667		23.7	43.9	
1922	ST078668		59.1		
1923	ST078669		43.3		
1924	ST078670		21.7		
1925	ST078671	0.8	45.1		
1926	ST078672	16.6	59.8		
1927	ST078673	49.0	54.6	42.0	
1928	ST078674	69.3	55.8		
1929	ST078675			45.4	41.2
1930	ST078676		8.9	67.0	61.4
1931	ST078677	52.6		44.5	
1932	ST078678		26.5		
1933	ST078679	3.3	23.5		

1934	ST078680			42.7	
1935	ST078681	14.5	46.0		
1936	ST078682		59.7	41.8	
1937	ST078683		51.1		40.4
1938	ST078684		67.3	46.1	46.0
1939	ST078685		73.2	40.4	
1940	ST078686		51.6		
1941	ST078687	3.1	30.3		
1942	ST078688		34.5		
1943	ST078689		54.0		
1944	ST078690	47.8	24.6		
1945	ST078691		26.5		72.2
1946	ST078692		32.0		54.6
1947	ST078693		42.0		
1948	ST078694	5.8	9.9		
1949	ST078696				
1950	ST078697		27.6	43.7	
1951	ST078698				
1952	ST078699				
1953	ST078700				
1954	ST078701		3.9		
1955	ST078702	45.6	52.1		
1956	ST078703	34.0	6.1		
1957	ST078704	11.2	14.5		
1958	ST078705	8.5	31.1		
1959	ST078706	23.9	18.6		
1960	ST078707	24.7	32.2		
1961	ST078708	24.3	67.7		63.4
1962	ST078709	9.8	81.6		54.3
1963	ST078710	34.9	63.2		
1964	ST078711	10.3	4.4		46.6
1965	ST078712	9.4	20.3		
1966	ST078713		58.1		
1967	ST078714		44.1		
1968	ST078715		22.4		57.0
1969	ST078790	79.0		55.3	62.1
1970	ST078813				44.0
1971	ST078878	2.8	16.3		
1972	ST078907	72.6			41.7
1973	ST078934	11.0			44.4
1974	ST079028		5.8		
1975	ST079153		14.4	71.9	
1976	ST079155		29.6		50.8
1977	ST079165			57.6	
1978	ST079167				56.8
1979	ST079280				68.8
1980	ST079282	90.9			60.7
1981	ST079284				
1982	ST079289	1.4	9.7		63.4
1983	ST079364		9.1		82.1
1984	ST079369		20.6		

1985	ST079453	59.7	18.7		
1986	ST079476		65.8		42.0
1987	ST079484	44.2	8.7		
1988	ST079485	15.9	10.9		
1989	ST079489	56.5	69.8		
1990	ST079495		19.1		
1991	ST079499				70.2
1992	ST079500	10.2	4.6		74.8
1993	ST079501		5.9	68.2	
1994	ST079502	94.3	8.0		70.7
1995	ST079503		17.8		
1996	ST079530	52.8	20.4		
1997	ST079544	39.6			
1998	ST079545	36.3			43.0
1999	ST079546	39.3	11.8		
2000	ST079547	26.9			63.5
2001	ST079548	7.6			
2002	ST079564	7.5		47.3	
2003	ST079565				
2004	ST079566	11.3	11.8		
2005	ST079567		17.4	60.9	
2006	ST079623	11.4	13.6		
2007	ST079624	19.2	6.0		
2008	ST079630	62.1	98.1		
2009	ST079631				
2010	ST079632				
2011	ST079633				
2012	ST079635		54.4		
2013	ST079636		36.8		
2014	ST079637		14.3		
2015	ST079638		28.9		
2016	ST079639	24.6	48.4		
2017	ST079640			42.5	
2018	ST079641		11.5		
2019	ST079642		22.6		
2020	ST079643		5.5		
2021	ST079644		35.3		
2022	ST079645		31.6		
2023	ST079646		21.1		
2024	ST079647	6.0	36.8		
2025	ST079648	13.9	35.3		
2026	ST079649	2.9	25.4		
2027	ST079650	19.4	23.4		
2028	ST079651	14.5	35.5		
2029	ST079652	7.6	13.8	78.3	
2030	ST079653		14.5	51.5	
2031	ST079654		28.5		
2032	ST079657	80.3	42.8	48.1	
2033	ST079659		27.3		
2034	ST079660		31.2		
2035	ST079726		27.3		

2036	ST079871	8.9	32.6		
2037	ST080029		24.6		
2038	ST080040	18.1	33.0		
2039	ST080041		50.0		
2040	ST080052	16.9	46.2	56.7	
2041	ST080056	40.7	45.8		
2042	ST080057	53.7	41.2		
2043	ST080074		26.6		
2044	ST080076		35.8	40.8	
2045	ST080077	40.8	44.8		
2046	ST080081		28.1		
2047	ST080082		42.1		
2048	ST080083	8.4	27.5		
2049	ST080087	0.8	11.9		
2050	ST080089	11.8	16.1		
2051	ST080090		35.2		
2052	ST080091	8.3	69.1		
2053	ST080092	2.5	12.2		
2054	ST080096	16.8	54.0		
2055	ST080105	40.8	15.5		
2056	ST080125	48.6	46.2		
2057	ST080126		26.8	49.1	
2058	ST080127		33.2	52.7	
2059	ST080128		34.9		
2060	ST080239		27.7		
2061	ST080343		16.7		
2062	ST080500		25.5		
2063	ST080581		7.3	41.7	
2064	ST080583		29.6	51.2	
2065	ST080592	54.1	48.7		
2066	ST080596	68.9	18.3		
2067	ST080640	23.3	61.7		
2068	ST080648	55.3	51.8		
2069	ST080679	88.7	97.0		
2070	ST080689		15.8		
2071	ST080919	11.1	34.3		
2072	ST080920	2.3	18.8		
2073	ST080922				
2074	ST080924	5.7	46.5		
2075	ST081095	5.4	69.1		
2076	ST081112		7.5		
2077	ST081146		27.6		
2078	ST081199		38.8		
2079	ST081307				
2080	ST081308		51.6		
2081	ST081309	7.1	32.4		
2082	ST081329		76.4		
2083	ST081332	69.1	91.0		
2084	ST081356	95.4	99.8	99.2	100.2
2085	ST081364	44.7	65.0		59.4
2086	ST081380	93.0	99.8	46.2	102.0

2087	ST081391		50.9	40.7
2088	ST082106		59.4	
2089	ST082211		23.0	
2090	ST082217	2.3	40.2	40.5
2091	ST082221	4.6	55.2	
2092	ST082228	26.1	51.3	
2093	ST082229	89.0	87.1	
2094	ST082231	18.6	55.6	
2095	ST082266		31.9	56.0
2096	ST082331	42.0	45.9	
2097	ST082475		30.1	
2098	ST082500	22.2	45.7	
2099	ST082525	6.9	36.5	
2100	ST082627	33.6	51.6	
2101	ST082628	23.3	40.7	
2102	ST082643	4.2	24.2	
2103	ST082651	2.8	52.8	46.1
2104	ST082661	75.3	44.2	
2105	ST082675	15.8	31.6	
2106	ST082678	16.9	45.9	
2107	ST082692	27.3	50.2	
2108	ST082719	14.7	38.6	
2109	ST082735	44.4	53.5	
2110	ST082824	46.9	35.9	
2111	ST083053	2.2	33.5	47.2
2112	ST083078	19.4	34.5	
2113	ST083088	23.0	27.8	46.8
2114	ST083089	27.9	48.3	
2115	ST083090	39.9	51.2	
2116	ST083091	10.1	42.1	43.4
2117	ST083092	101.8	101.9	
2118	ST083093	67.5	101.4	
2119	ST083110	5.0	48.4	
2120	ST083122	30.5	57.1	59.8
2121	ST083127		3.3	46.2
2122	ST083128	32.3	51.5	50.5
2123	ST083129	12.7	28.4	
2124	ST083130	24.1	50.0	
2125	ST083131	9.7	34.1	
2126	ST083132	47.4	89.4	
2127	ST083133	20.1	51.3	
2128	ST083134	22.7	48.2	
2129	ST083256		55.0	
2130	ST083266		44.8	40.9
2131	ST083274	4.7	36.2	
2132	ST083302		43.4	
2133	ST083484	19.9	50.5	
2134	ST083487	11.9	44.5	
2135	ST083581	4.7	45.2	
2136	ST083600		38.5	56.0
2137	ST083605	17.6	50.2	

2138	ST083608	7.9	31.4		
2139	ST083626		33.6		
2140	ST083629	8.3	49.2		
2141	ST083675	45.8	52.9		
2142	ST083836	45.7	39.7		
2143	ST083912	13.0	34.0		
2144	ST084047	18.3	53.0		
2145	ST084404	2.7	34.8		
2146	ST084741	12.8	29.5		
2147	ST084802	11.8	37.8		
2148	ST084836	18.3	41.3		
2149	ST085104	42.5	88.3	63.7	
2150	ST085493	24.3	38.4		
2151	ST085494	71.0	85.4		
2152	ST085666	29.8	38.5	42.1	
2153	ST085667		46.0	63.3	
2154	ST085670	34.0	32.4	49.5	
2155	ST085671	69.4	86.5		
2156	ST085757		33.1	46.5	
2157	ST085760		36.6	40.9	
2158	ST085763	44.7	27.2		
2159	ST085775	25.5	38.6		
2160	ST085783	53.8	48.7		
2161	ST085788		41.7		45.6
2162	ST085789		14.6	49.6	
2163	ST085801		0.3		42.3
2164	ST085807	77.8	86.4		
2165	ST085820	4.8	25.2		
2166	ST085825	5.9	12.3	53.4	
2167	ST085828	16.0	14.0		
2168	ST085831	27.8	38.9		
2169	ST085868		17.1		
2170	ST085869	31.2	35.8		
2171	ST085906	20.4	41.5		
2172	ST085907		40.6		
2173	ST085913	5.1	25.3		
2174	ST085917	11.8	11.6		
2175	ST085923	42.7	39.0		
2176	ST086044	21.2	78.1		
2177	ST086048		15.3		
2178	ST086086		19.6		
2179	ST086088		46.7		
2180	ST086090		43.3		
2181	ST086091		22.7		
2182	ST086092	25.3	66.4		
2183	ST086108		18.3		
2184	ST086116	77.8	59.5		
2185	ST086117		10.0		
2186	ST086165	30.7	31.4		
2187	ST086169		3.5		
2188	ST086226		6.4		

2189	ST086230				
2190	ST086238	31.6	31.5		
2191	ST086243	94.1	85.8		
2192	ST086259	23.5	24.8		
2193	ST086260	30.6	34.7		
2194	ST086315	3.7			
2195	ST086322	14.2	37.2		
2196	ST086350		35.2		
2197	ST086398	1.6	24.4		
2198	ST086401	5.8	7.4		
2199	ST086404	40.9	22.9		
2200	ST086422	34.8	46.3	60.9	
2201	ST086476	11.1	65.5		
2202	ST086477		0.2		
2203	ST086480		41.9		
2204	ST086485		8.6		
2205	ST086488		42.0		
2206	ST086489	92.5	92.4	103.7	105.5
2207	ST086494	14.1	41.2		
2208	ST086496		35.0		
2209	ST086501				
2210	ST086505		59.0		
2211	ST086509		1.3		
2212	ST086510		14.4		
2213	ST086511	90.5	71.2		
2214	ST086512		39.0		
2215	ST086513		15.6		
2216	ST086530	16.8	62.2		
2217	ST086541		53.5		
2218	ST086591				
2219	ST086601	8.2	39.5		
2220	ST086620	56.5	30.1		
2221	ST086622	55.3	74.9		
2222	ST086712				
2223	ST086721	5.8	16.4		
2224	ST086741	27.5	55.6		
2225	ST086748	2.1	13.3		
2226	ST086886	21.9	56.0		
2227	ST086887	14.3	54.3		
2228	ST086948	10.5	30.9		
2229	ST086949	12.6	47.9		
2230	ST086950	55.7	72.6		
2231	ST086951	9.5	47.5		
2232	ST086964	17.2	56.2		
2233	ST086965	9.9	77.5		
2234	ST086966		60.0		
2235	ST086967		44.1		
2236	ST086968		35.7		
2237	ST086969	9.6	63.2		
2238	ST086988				
2239	ST087014	88.2	58.4		

2240	ST087015	78.1	61.1		
2241	ST087016	57.9	68.9		
2242	ST087017	83.8	79.5		
2243	ST087018	78.0	64.1		
2244	ST087021		49.4		
2245	ST087031		36.4		
2246	ST087034	9.1	52.6		
2247	ST087043	28.5	62.6		
2248	ST087045		5.4		
2249	ST087062				
2250	ST087363				
2251	ST087792		43.0		
2252	ST087906		22.3		
2253	ST087910		47.0		
2254	ST087911		32.9		
2255	ST087915		21.5		
2256	ST087942	84.4	44.8		
2257	ST087943	63.3	55.1		
2258	ST087945	82.6	81.9		
2259	ST087947	92.8	83.1		
2260	ST087949	37.4			
2261	ST087968		35.3		
2262	ST087977				
2263	ST087979		36.1		
2264	ST087981	3.8	21.5		
2265	ST088013	49.9	50.2		
2266	ST088015		40.6		
2267	ST088036		17.7		
2268	ST088039		34.9		
2269	ST088060		7.2		
2270	ST088071		28.8		
2271	ST088072		41.1		
2272	ST088074		61.3	46.7	
2273	ST088080		21.8	40.3	
2274	ST088107		42.8		
2275	ST088110	19.1	60.7		
2276	ST088111		12.2		
2277	ST088169		23.6		
2278	ST088170	20.6	54.2		
2279	ST088176	58.4	61.8		
2280	ST088177	96.2	94.4	98.1	99.1
2281	ST088185		18.2		
2282	ST088217	46.0	41.6		
2283	ST088220				
2284	ST088330				
2285	ST088333	93.4	99.0	99.0	99.3
2286	ST088370		33.3		
2287	ST088379		4.9		
2288	ST088380		19.5		
2289	ST088381		37.9		
2290	ST088382		31.2	44.9	

2291	ST088393	80.1	66.4		
2292	ST088394	85.5	75.1		
2293	ST088395	93.9	83.9		
2294	ST088396	49.1	54.6		
2295	ST088397	62.3	48.5		
2296	ST088398	87.8	62.1		
2297	ST088399	87.3	86.6		
2298	ST088400	48.2	37.8		
2299	ST088401	94.6	87.1		
2300	ST088408		1.6		
2301	ST088409		12.4		
2302	ST088410				
2303	ST088411		19.5		
2304	ST088414		10.2		
2305	ST088437		58.2		
2306	ST088525				
2307	ST088527	98.8	98.6		
2308	ST088558		7.8		
2309	ST088560		51.7		
2310	ST088565	64.5	66.3		
2311	ST088566	61.8	80.5		
2312	ST088567	90.7	73.0		
2313	ST088568		26.2		
2314	ST088569		24.0		
2315	ST088588		35.6		
2316	ST088591		13.9		
2317	ST088594		25.8		
2318	ST088596		30.3		
2319	ST088597		23.5		
2320	ST088598	18.3	16.2		
2321	ST088600	10.5			56.6
2322	ST088601	22.0	11.6		
2323	ST088602		28.4		45.5
2324	ST088606		23.8		98.5
2325	ST088608		23.6		101.3
2326	ST088612	3.5	24.9	51.0	
2327	ST088623	12.2	60.8		
2328	ST088626	7.8	51.6		
2329	ST088627	2.9	11.5		98.1
2330	ST088628	55.3	34.5		
2331	ST088633		2.3		
2332	ST088642		8.1		
2333	ST088645		7.1		
2334	ST088646			45.8	
2335	ST088654		12.6		
2336	ST088656	18.5	29.9		
2337	ST088657	15.0	25.9		
2338	ST088675	62.4			
2339	ST088690	18.5	10.0		
2340	ST088703	11.9	27.6		
2341	ST089008		12.3	46.5	

2342	ST089011				
2343	ST089016		12.9		
2344	ST089287	6.5	10.6		
2345	ST089303	37.9	26.6		
2346	ST089417	88.7	64.5		
2347	ST089418	59.3	45.4		
2348	ST089419		33.8		
2349	ST089420	16.9	8.3		
2350	ST090280	22.0	13.6		
2351	ST090282	28.5	15.4		
2352	ST090286	20.0			
2353	ST090287		5.6		
2354	ST090292	67.4	12.3		
2355	ST090299	23.1	17.8		
2356	ST090302	22.7	21.7		
2357	ST090304	16.8			
2358	ST090308	16.3	2.3		
2359	ST090309		5.5		
2360	ST090310	5.5	10.4		
2361	ST090311	53.5	1.4		
2362	ST090475	29.3	35.1	43.4	
2363	ST092008	35.4	26.0		
2364	ST092266	32.4	2.1		
2365	ST092267	28.6	48.2		
2366	ST092285	62.9	45.3		
2367	ST092286			53.6	
2368	ST092287	9.0	10.0	42.6	
2369	ST092291				
2370	ST092292	51.6	7.7		
2371	ST092293	85.7	95.3		
2372	ST092296	18.3	91.1		
2373	ST092297	18.9			
2374	ST092300	7.3	4.5		
2375	ST092303	3.1	15.8		
2376	ST092306	20.7	12.6		
2377	ST092309		16.2		
2378	ST092311	11.7	4.8		
2379	ST092317	0.4			
2380	ST092328	3.1			
2381	ST092330	15.2			
2382	ST092332				
2383	ST092333	17.5			
2384	ST092334	35.5			
2385	ST092335	7.3	15.3		98.2
2386	ST092337	20.0	19.0		
2387	ST092338		18.4		
2388	ST092339		22.0		
2389	ST092340	102.5	97.7	94.1	
2390	ST092341		18.9		
2391	ST092343	24.5	5.0		
2392	ST092355		27.2		

2393	ST092357	30.7			
2394	ST092359		17.6		
2395	ST092360	7.5	22.9		
2396	ST092364		24.2		
2397	ST092368	11.1	7.2		
2398	ST092369		13.8		
2399	ST092370		13.1	48.4	
2400	ST092373		0.7		
2401	ST092511	35.2	17.9		
2402	ST092512	26.4	18.7		
2403	ST092513			49.9	
2404	ST092552	24.8	49.8		
2405	ST092571				
2406	ST092572	16.6			
2407	ST092582	71.5	43.2		
2408	ST092584	62.5	5.8		
2409	ST092585	78.5	42.3	112.3	92.1
2410	ST092586	71.2	25.0		
2411	ST092587	57.3	0.8		
2412	ST092601				
2413	ST092612	18.6	8.5		
2414	ST092622	6.3			
2415	ST092632	9.1	50.7		
2416	ST092652	22.4	18.9		
2417	ST092660			112.3	117.8
2418	ST092677			42.3	
2419	ST092685				
2420	ST092688				
2421	ST092693				
2422	ST092696		69.0		
2423	ST092700		74.7		99.6
2424	ST092701		2.5	44.3	
2425	ST092702	27.1			
2426	ST092703	12.4			
2427	ST092704			47.3	
2428	ST092705				47.0
2429	ST092706	12.1	1.0		
2430	ST092707	10.1	0.1		
2431	ST092708	22.4	14.2		
2432	ST092710	10.5	41.4		
2433	ST092714	46.2	12.0		
2434	ST092719	12.6			
2435	ST092727	1.6			
2436	ST092737		3.7		
2437	ST092763				
2438	ST092764			51.1	
2439	ST092777			44.4	
2440	ST092778	42.9		43.3	
2441	ST092779	11.7		42.8	
2442	ST092780	12.8		68.2	
2443	ST092781			58.6	

2444	ST092782	4.8	4.8	61.2	
2445	ST092784		6.8	68.9	
2446	ST092785		8.4	40.9	
2447	ST092786		7.0		
2448	ST092787	1.2	22.0		
2449	ST092790	25.1			
2450	ST092791	56.1	16.8		
2451	ST092792				
2452	ST092793	15.8	58.9	59.5	
2453	ST092794		31.2	42.6	
2454	ST092795				
2455	ST092796	18.3	19.7		
2456	ST092823	71.3	62.9		
2457	ST092832			112.3	
2458	ST092833			53.0	
2459	ST092848				
2460	ST092850	50.0	20.5		
2461	ST092857	15.5	13.9		
2462	ST092863			66.7	
2463	ST092866		6.3	60.9	
2464	ST092870	39.9		52.0	
2465	ST092936			112.3	117.0
2466	ST092942	49.4	23.9		
2467	ST092966		32.3		
2468	ST092971	58.2	37.7	102.7	
2469	ST092972		55.0	87.6	
2470	ST093057	88.4	78.0	77.5	
2471	ST093063			50.6	46.1
2472	ST093084		107.2	48.6	
2473	ST093395			50.2	
2474	ST093424				
2475	ST093460				
2476	ST093461				
2477	ST093489				
2478	ST093526				
2479	ST093529				
2480	ST093534			55.8	
2481	ST093540	98.9	78.9	94.6	64.8
2482	ST093559				44.3
2483	ST093567	18.4	1.3		
2484	ST093576				
2485	ST093597	89.1	59.4		
2486	ST093631		1.1		
2487	ST093641			40.2	
2488	ST093686	33.6	35.7		
2489	ST093687	33.2			
2490	ST093688				
2491	ST093689				
2492	ST093690				
2493	ST093691				
2494	ST093693				

2495	ST093694				
2496	ST093695	59.3	43.4		53.8
2497	ST093698	13.2			86.4
2498	ST093700				
2499	ST093702	37.7	82.6		
2500	ST094679			42.0	
2501	ST094685	6.6			
2502	ST094732	13.9	7.2		
2503	ST094739			56.2	
2504	ST094749	1.5			
2505	ST094802				
2506	ST094804				
2507	ST094812	25.6			
2508	ST094815				
2509	ST094816	4.5			
2510	ST094817				
2511	ST094818	32.8			
2512	ST094820	55.7	1.1		
2513	ST094821				
2514	ST094822				
2515	ST094823				
2516	ST094824				
2517	ST094825				
2518	ST094828				
2519	ST094829				
2520	ST094832				
2521	ST094835		46.7		
2522	ST094845		7.0		
2523	ST094856		19.8		
2524	ST094858				
2525	ST094874		16.4		
2526	ST094877	18.7	45.3		
2527	ST094894		17.6		
2528	ST094936	7.3	25.6		
2529	ST094937	0.5			
2530	ST094938				
2531	ST094951				
2532	ST094971		12.2		
2533	ST094972	36.2	42.1		
2534	ST094973	34.2	43.8		
2535	ST094981	109.8	110.0	97.8	88.1
2536	ST095092	81.3	35.8		
2537	ST095103				68.1
2538	ST095117		35.0		
2539	ST095118		32.0		
2540	ST095119	13.5	50.1		
2541	ST095120		29.8		
2542	ST095121		50.5		
2543	ST095122	17.4	37.6		
2544	ST095123	5.2	41.5		
2545	ST095124	20.2			111.8

2546	ST095125				
2547	ST095126				
2548	ST095127		21.5		
2549	ST095128		31.0	55.6	
2550	ST095129				
2551	ST095130		7.9		
2552	ST095132				
2553	ST095133				
2554	ST095134				
2555	ST095135				
2556	ST095141	17.1			
2557	ST095142				
2558	ST095143				
2559	ST090257	16.5			
2560	ST095170	55.6	28.5		
2561	ST095176				
2562	ST095187	76.0	58.9		
2563	ST095189	105.7	76.5		
2564	ST095190	47.2			
2565	ST095191	52.6			
2566	ST095192	55.6		55.7	
2567	ST095193	58.3		48.0	
2568	ST095194	60.9	31.1	60.9	
2569	ST095195		8.9		
2570	ST095196	11.4	12.7	64.4	40.3
2571	ST095197	38.5			
2572	ST095198	28.9		66.2	37.2
2573	ST095199	64.1			
2574	ST095200	44.4			37.9
2575	ST095201	43.0			
2576	ST095202	63.7	105.5	60.7	
2577	ST095206			113.1	
2578	ST095207	32.8		54.6	
2579	ST095208	40.6		51.8	
2580	ST095209	35.0		63.0	
2581	ST095210	43.5			
2582	ST095212	107.4	33.3	56.7	
2583	ST095283	26.4		75.3	
2584	ST095355	20.5	106.4		
2585	ST095378			88.4	
2586	ST095398	102.2	91.9	61.1	
2587	ST095408	22.0	49.7		
2588	ST095409	21.2	18.9	45.9	
2589	ST095410	13.3		58.5	
2590	ST095411	119.1	73.4	100.2	100.6
2591	ST095412	87.1	83.8		
2592	ST095413	106.6	76.1	75.5	100.2
2593	ST095414	57.2	61.0		83.6
2594	ST095416	21.2	21.6		
2595	ST095417	113.5	77.7	97.3	102.3
2596	ST095418	112.2	85.1	99.6	101.2

2597	ST095419				
2598	ST095420	28.2			
2599	ST095443		25.6	54.4	
2600	ST095447	40.7	25.5	42.7	
2601	ST095471	83.8	60.3	60.3	
2602	ST095475	54.8		41.4	
2603	ST095499	52.1	4.5		
2604	ST095588	50.8	11.8		
2605	ST095590	64.3	28.7		
2606	ST095607	59.6	5.5		
2607	ST095617	59.7	38.6		
2608	ST095620	110.3	95.2	98.1	83.9
2609	ST095636		33.2	64.1	
2610	ST095638	14.8			
2611	ST095639	4.9	30.9		
2612	ST095656		55.1	55.0	
2613	ST095663	48.0	62.5	50.3	
2614	ST095687		30.2		
2615	ST095688	22.0	64.5		
2616	ST095697	48.8	55.1	48.4	
2617	ST095706		17.6		
2618	ST095712		15.1		
2619	ST095719	27.0	26.1		
2620	ST095727	33.0	51.6		
2621	ST095729		37.6		
2622	ST095736	16.2	35.0		
2623	ST095747		50.9		
2624	ST095754		78.1		
2625	ST095759		72.5		
2626	ST095762	60.0			
2627	ST095765			44.6	
2628	ST095769	0.4			
2629	ST095784			51.3	
2630	ST095787	103.3	85.3	99.4	
2631	ST095788	100.8	90.3	100.3	99.6
2632	ST095790	12.4	106.1		
2633	ST095791	52.9			
2634	ST095812	17.9			
2635	ST095820	60.2			
2636	ST000096				
2637	ST001576	2.8		53.4	
2638	ST001581	55.3			
2639	ST001592	8.2	15.9	63.0	
2640	ST001801	60.2	39.2		
2641	ST001804	12.7	72.3		
2642	ST002037		37.7		
2643	ST002230		44.0		
2644	ST002253		40.9		
2645	ST002382		60.2		
2646	ST002428		54.5		
2647	ST003500		53.7		

2648	ST003847		58.9	82.4	
2649	ST003849		41.1		122.5
2650	ST004308		2.6		
2651	ST004332		83.2		
2652	ST004647		18.6		
2653	ST004867		13.6		
2654	ST005060		32.1		
2655	ST005061		29.2		
2656	ST005181	112.0	60.7		
2657	ST005184		43.1		
2658	ST005187		26.4		
2659	ST005192		59.1		
2660	ST005196		48.4		
2661	ST005198		42.3		
2662	ST005212		51.3		
2663	ST005315		56.4		
2664	ST005480	110.5	81.0		
2665	ST006023		28.6		
2666	ST007136		90.8		
2667	ST007254		65.7		
2668	ST008188		50.8		
2669	ST008697		59.5		
2670	ST095428		33.0		
2671	ST008976		26.5		
2672	ST008977		47.7	88.3	
2673	ST012361	15.5	42.5		
2674	ST012879	10.7			
2675	ST012880	90.0	9.9		
2676	ST012881	11.9	40.5		
2677	ST012884	28.6	51.7	45.1	
2678	ST012890		40.7		
2679	ST015023		40.5		
2680	ST018286		62.8		
2681	ST018578		19.8		
2682	ST019440		44.0		
2683	ST019450	53.2	68.8		
2684	ST020193		62.7		
2685	ST020194		60.1		
2686	ST020197		45.8		
2687	ST020200		38.7		
2688	ST022011		41.4		
2689	ST022313		48.5		
2690	ST023605		27.5		47.2
2691	ST023722		27.6		
2692	ST024011		34.3		
2693	ST024013		58.4		
2694	ST024026		49.1		
2695	ST024062		60.4		
2696	ST024087	14.9	82.7	47.7	
2697	ST024095		34.0	58.8	123.2
2698	ST024096	13.4		46.5	

2699	ST025083	57.3	24.0		
2700	ST025101	37.1	27.1		
2701	ST025283	21.0	62.8		
2702	ST025320	28.6	39.4		
2703	ST025422	114.0	35.8		
2704	ST026558	111.3	62.2		
2705	ST026972		22.1		123.1
2706	ST026983		12.5		
2707	ST028983		50.7		
2708	ST031675		21.1		
2709	ST032128	9.8	47.0		
2710	ST032142		47.4		
2711	ST033593		47.4	43.3	
2712	ST034304		106.2		
2713	ST034305		51.9	42.1	
2714	ST034307	33.3	22.6		
2715	ST034308	68.2	61.4		
2716	ST034309	45.1	20.0		
2717	ST034310	64.7	67.2		
2718	ST037430	39.5	33.1		
2719	ST037662	30.1	40.9		
2720	ST038384	108.2	59.3		
2721	ST038611	44.9	65.7		
2722	ST038698	50.0	69.7	41.0	
2723	ST038710	27.7	71.7		
2724	ST038711	70.8	72.4	56.4	
2725	ST038788	69.2	58.0		
2726	ST038790	43.6	61.3		
2727	ST038804	38.3	76.6	67.2	
2728	ST039159	71.4	64.8		
2729	ST039254	46.7	82.8		
2730	ST041685	55.2	73.0		
2731	ST041785	25.7	62.8		
2732	ST041790	18.1	55.3		
2733	ST041812		53.5		
2734	ST041828	30.8	53.7	61.8	
2735	ST041867	30.9	57.4		
2736	ST041868	90.5	63.2		
2737	ST041875	44.7	86.6		
2738	ST042005	77.5	97.1		
2739	ST043095	22.4	54.3		
2740	ST044296	25.5	49.9		
2741	ST044297	27.8	34.8	57.0	
2742	ST044904		48.8		
2743	ST045055	38.0	35.8		
2744	ST045201	82.1	30.7		
2745	ST045278	77.0	60.5		
2746	ST045447	20.8	48.2		
2747	ST045756	43.3	53.0		
2748	ST045815	32.5	40.5		
2749	ST045861	42.5	51.7		

2750	ST045862	59.6	43.7		
2751	ST045910	88.9	43.9		
2752	ST046012	109.9	33.8		
2753	ST046180	67.4	80.3		
2754	ST046819	21.2	81.2		
2755	ST046998	30.9	61.4		
2756	ST047698	43.8	71.7		
2757	ST047810	20.5	34.2		
2758	ST047895	58.9	87.1		
2759	ST048085	17.9	70.5		
2760	ST048321	103.7	85.2	98.2	88.2
2761	ST048695	61.8	102.3		
2762	ST049570	8.9	81.8		
2763	ST050139	35.0	75.3		
2764	ST050168	42.7	84.8		
2765	ST050382	19.7	62.9		
2766	ST050825		62.2		
2767	ST050890		70.2		
2768	ST051122	20.7	76.4	40.7	
2769	ST051125	44.8	66.5		
2770	ST051508	54.3	64.9		
2771	ST051621	15.6	49.9		
2772	ST051725	35.6	60.6		
2773	ST051792	48.9	44.1		
2774	ST051832	33.3	57.4	44.1	
2775	ST051891	17.5	76.3		
2776	ST052085	31.9	52.6		
2777	ST053504	13.2	74.3		115.2
2778	ST053665		60.8		
2779	ST053879	2.6	61.2		
2780	ST055531	45.5	52.8		
2781	ST056203	35.8	80.4		
2782	ST056930	103.0	94.4	53.1	85.5
2783	ST057112	75.8	64.5		
2784	ST057647	90.5	80.2		
2785	ST058412	23.2	86.9		
2786	ST058451	12.0	32.2		
2787	ST059872	25.1	26.0		
2788	ST059957	27.9	35.5		
2789	ST060822	40.9	27.6		
2790	ST062138	33.7	17.5		
2791	ST063098	29.4			
2792	ST064877	29.0	30.3		
2793	ST067135	63.9	83.4		73.1
2794	ST067355	30.6	78.3		
2795	ST067672	62.5	43.8		
2796	ST067700	67.6	53.9		
2797	ST067711	44.5	48.7		
2798	ST068062	72.3	38.4		
2799	ST068261	30.7	27.0		
2800	ST068262	55.3	27.2		

2801	ST068290		9.2		
2802	ST068357				
2803	ST068825				
2804	ST069390				
2805	ST069392	3.8			
2806	ST069448			58.7	
2807	ST069459				
2808	ST070482			77.1	58.4
2809	ST070784		68.4		
2810	ST070948	12.3			
2811	ST070950	45.6	39.3	54.0	
2812	ST070964				
2813	ST070969	4.0			
2814	ST070975				
2815	ST072434		4.5	48.3	46.0
2816	ST072574	23.7	21.8	108.4	125.9
2817	ST072626		41.4		
2818	ST072632				
2819	ST072644				
2820	ST072674				
2821	ST072700				
2822	ST072715				
2823	ST072754	53.9	10.7		
2824	ST072771	56.5	12.1	112.5	
2825	ST072787	38.3	16.1		
2826	ST072789				
2827	ST072790				
2828	ST072837				
2829	ST072853	11.4			
2830	ST073199				
2831	ST073264				
2832	ST073304	6.7	22.3		
2833	ST073324	56.1	18.3		
2834	ST073344				
2835	ST073377				
2836	ST073411				
2837	ST073523				
2838	ST073552				
2839	ST073598	13.0			
2840	ST073708	21.5			
2841	ST073730	6.4	15.3		
2842	ST073732				
2843	ST073735			50.5	
2844	ST073808		62.5	84.9	65.1
2845	ST073935				
2846	ST074588	23.6			
2847	ST074621				
2848	ST074622				
2849	ST074623	1.5			
2850	ST074626				
2851	ST074899				

2852	ST074924				
2853	ST074977				
2854	ST074982				
2855	ST075216	40.8			
2856	ST075219		19.5		
2857	ST075227	39.4			
2858	ST075585			46.2	
2859	ST075586				
2860	ST075616				
2861	ST075617				
2862	ST075618				
2863	ST075646				
2864	ST076085	3.7		84.9	
2865	ST076773	1.5		73.0	
2866	ST076961			53.6	
2867	ST076964			69.0	
2868	ST077033	46.3	10.0		
2869	ST077035				
2870	ST077129				
2871	ST077137			57.1	
2872	ST077139	38.2	53.0	76.0	
2873	ST077813		3.4		
2874	ST077819		9.6		
2875	ST077846				
2876	ST077849	6.3			
2877	ST078135	44.8	5.7	91.8	59.9
2878	ST078149				
2879	ST078159			45.9	
2880	ST078887			61.0	
2881	ST079060	6.3			
2882	ST079063	4.9	78.9		
2883	ST079066	39.0	73.9		
2884	ST079081	37.5	32.3		
2885	ST079085	5.1	82.0		
2886	ST079100	5.2	36.2		
2887	ST079107		1.2		
2888	ST079110	35.0	34.2		
2889	ST079112				
2890	ST079113		24.3		
2891	ST079117		27.1		
2892	ST079123		27.1		
2893	ST079127	0.9	16.1		
2894	ST079134		55.4		
2895	ST079689	8.9	38.7		
2896	ST079962	94.5	91.2		
2897	ST080013	6.0	82.1		
2898	ST080046	3.0	9.7		
2899	ST080093	16.8	56.2		
2900	ST080605	7.3	68.6		
2901	ST080893	40.2	98.4		
2902	ST081445	41.6	73.0		

2903	ST081447	43.8	58.3		
2904	ST081450	86.4	98.0		
2905	ST081454	37.9	28.7		
2906	ST081455	27.6	13.7		
2907	ST081457	11.6			
2908	ST081459	46.0	76.7		
2909	ST081483	12.1	99.9		
2910	ST081488	26.4	25.5		
2911	ST081490	59.6	54.8		
2912	ST081565	13.0	31.6		
2913	ST081583	52.9	37.4		
2914	ST081608	34.7	86.1		
2915	ST081613	74.9	71.9		
2916	ST081723	19.7	98.3		
2917	ST082265	13.7	89.7		
2918	ST082303	22.4	35.5		
2919	ST082304	43.2	57.4		
2920	ST082306	94.8	38.6		
2921	ST082314	13.3			
2922	ST082323	36.6	21.5		
2923	ST082327	59.6	45.8		
2924	ST082429	11.2	29.3		
2925	ST082848	35.4	56.6		
2926	ST082886	17.8	42.8		
2927	ST083082	13.2	66.8		
2928	ST083084	14.2	21.8		
2929	ST083085	11.2	60.6		
2930	ST083391	72.8	74.7		
2931	ST083392	12.5	0.4		
2932	ST083398	28.0	9.3		
2933	ST083439	36.1	30.0		
2934	ST083715	31.5	42.1		
2935	ST083850	7.2	49.1		
2936	ST083877	19.5			
2937	ST083914	74.0	19.8		
2938	ST083925	17.3	31.0		
2939	ST084063		33.2		
2940	ST084155	10.4	38.3		
2941	ST084235	48.6	61.1		
2942	ST084271	18.1	41.8		
2943	ST084423	24.1	69.7		
2944	ST084442	97.7	95.8	101.0	64.7
2945	ST084458	28.0	14.5		
2946	ST084469	86.9	91.9	62.7	
2947	ST084474	2.0	35.1		
2948	ST084517	17.9	72.6		
2949	ST084526	42.6	101.0		
2950	ST084623		36.2		
2951	ST084658	19.6	65.8		
2952	ST084742	47.7	33.9		
2953	ST084926	57.4	21.8		

2954	ST084956	18.5	29.9		
2955	ST085054	14.3	34.9		
2956	ST085098	52.6	41.3		
2957	ST085304	78.6			
2958	ST085314	81.7	89.9		
2959	ST085342	25.5	11.8		
2960	ST085343	15.6	39.0		
2961	ST085502	41.9	36.8		56.6
2962	ST085508	26.7	45.7		
2963	ST085623	2.8		40.4	45.5
2964	ST085684	98.6	95.4		98.5
2965	ST085690	96.1	82.0	83.5	101.3
2966	ST085855	54.0	55.0		
2967	ST085897	92.7	69.1		
2968	ST085899	90.1	84.6		
2969	ST085955	97.0	96.9	98.3	98.1
2970	ST086020	54.0	83.7		
2971	ST086022	35.5	78.7		
2972	ST086025		70.3		
2973	ST086026	40.4			
2974	ST086028		47.9		
2975	ST086041		53.5		
2976	ST086527		34.7		
2977	ST086528	32.5	67.6		
2978	ST086529		29.8		
2979	ST086531	14.3	59.8		
2980	ST086532		61.2		
2981	ST086533		41.2		
2982	ST086534	74.8	95.1		
2983	ST086538	62.7	74.0		
2984	ST086539	11.8	47.3		
2985	ST086540	22.8	65.8		
2986	ST086542		54.1		
2987	ST086543		52.6		
2988	ST086544	19.0	46.2		
2989	ST086545	8.4	44.5		
2990	ST086563	52.1	79.8		
2991	ST086627	9.3	65.2		
2992	ST086881		65.2		
2993	ST086882		19.3		
2994	ST087186		31.6		
2995	ST088307		38.6	40.1	
2996	ST088496	9.3	40.1		
2997	ST088501		52.0		
2998	ST088502	6.1	80.2		
2999	ST088503		89.2	52.7	
3000	ST088505	13.9	38.2	41.7	
3001	ST088506	20.6	48.5		
3002	ST088508		33.6		
3003	ST088509		51.9		
3004	ST088515		54.3		

3005	ST088771	12.0	55.1		
3006	ST088843		72.9		
3007	ST088851		20.1		
3008	ST088858	32.3	59.1		
3009	ST088864	46.6	67.6		
3010	ST088865	39.5	61.7		
3011	ST088869	44.1	37.3		
3012	ST088881		55.8		
3013	ST088882		59.1		
3014	ST088922		55.9		
3015	ST088925		41.0	41.8	
3016	ST088939	3.5	59.5		
3017	ST088940	25.1	74.4		
3018	ST088945	16.7	66.0		
3019	ST088947	59.7	62.2		
3020	ST088948		62.1		
3021	ST088949	52.4	68.6		
3022	ST088966	21.4	61.8		
3023	ST088973	12.2	54.3		
3024	ST088975	37.6	56.7		
3025	ST088977	89.9	98.6	58.1	98.2
3026	ST088994	4.9	2.8		
3027	ST088996	33.9	9.7		
3028	ST088998		35.6	43.0	
3029	ST089001	43.7	56.5		
3030	ST089053	30.9			
3031	ST089073	9.3	7.9		
3032	ST089108	54.3			
3033	ST089109	33.7	64.9		
3034	ST089117	26.6	65.4		
3035	ST089133	43.0	52.3		
3036	ST089209		52.6	44.0	
3037	ST090252	65.1	76.7		
3038	ST090254	47.5	73.4		
3039	ST090255	8.4	47.7		
3040	ST090256	51.4			
Que		99.7	98.9		

Supplementary table 4. Compound codes and IUPAC names of the highly active compounds identified in primary screening.

Cpd	Compound code (supplier) and IUPAC name
**144**	**ST012391**<br>8-(4-amino-2-oxohydropyrimidinyl)-3-hydroxy-6-(hydroxymethyl)-2,4,7-trioxa-3-phosphabicyclo[3.3.0]octan-3-one
**339**	**ST024776**<br>(2S,5R,6R)-3,3-dimethyl-7-oxo-6-(2-phenylacetylamino)-4-thia-1-azabicyclo[3.2. 0]heptane-2-carboxylic acid, {2-[(4-aminophenyl)methoxy]ethyl}diethylamine, hydrate
**341**	**ST024784**<br>2-[1-(bromomethyl)vinyl]-8,9-dimethoxy-1,2-dihydrochromano[3,4-b]furano[2,3-h]chroman-6-one
**575**	**ST045414**<br>6-chloro-3-hydroxy-2-(3-pyridyl)chromen-4-one
**916**	**ST069294**<br>6,7-dihydroxy-2-phenylchromen-4-one
**1698**	**ST077117**<br>2-[(6-{4-[(6S,1R,5R)-6-(4-hydroxy-3-methoxyphenyl)-3,7-dioxabicyclo[3.3.0]oct-2-yl]-2-methoxyphenoxy}-3,4,5-trihydroxy(2H-3,4,5,6-tetrahydropyran-2-yl))methoxy]-6-methyl-2H-3,4,5,6-tetrahydropyran-3,4,5-triol
**2117**	**ST083092**<br>2-(2-chlorophenyl)-4-oxochromen-3-yl propanoate
**2307**	**ST088527**<br>4-indol-3-yl-5-methyl-1,3-thiazole-2-ylamine
**2896**	**ST079962**<br>2-(4-methoxyphenyl)-4-oxochromen-3-yl decanoate

Supplementary figure 1. Effects of the lead compounds (at 100 and 400 μM) on bacterial growth after 24 hours of incubation. Results are presented as mean ± SD, n = 3.

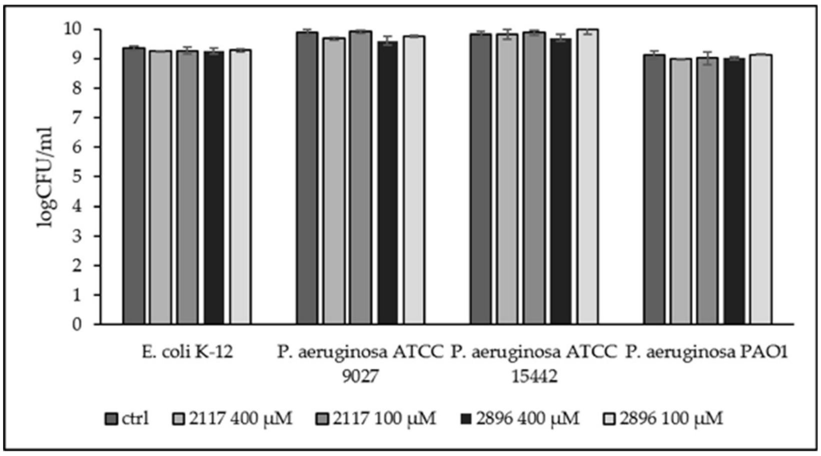

Supplement: Supplementary file 1 [file ijms-19-01346-s001.zip › Supplementary information (all except Supplementary table 3).pdf]
